# Circulating Tumour Cells Indicate the Presence of Residual Disease Post-Castration in Prostate Cancer Patient-Derived Xenograft Models

**DOI:** 10.3389/fcell.2022.858013

**Published:** 2022-04-13

**Authors:** Sara Hassan, Tony Blick, Jack Wood, Erik W. Thompson, Elizabeth D. Williams

**Affiliations:** ^1^ Queensland University of Technology (QUT), Faculty of Health, School of Biomedical Sciences at Translational Research Institute (TRI), Brisbane, QLD, Australia; ^2^ Australian Prostate Cancer Research Centre, Queensland (APCRC-Q) and Queensland Bladder Cancer Initiative (QBCI), Brisbane, QLD, Australia

**Keywords:** kallikrein related peptidase 3, metastasis, circulating tumour cell, castrate-resistant prostate cancer, epithelial mesenchymal plasticity, prostate specific antigen, patient-derived xenograft

## Abstract

Castrate-resistant prostate cancer (CRPC) is the lethal form of prostate cancer. Epithelial mesenchymal plasticity (EMP) has been associated with disease progression to CRPC, and prostate cancer therapies targeting the androgen signalling axis, including androgen deprivation therapy (ADT), promote EMP. We explored effects of castration on EMP in the tumours and circulating tumour cells (CTCs) of patient-derived xenograft (PDX)-bearing castrated mice using human-specific RT-qPCR assays and immunocytochemistry. Expression of prostate epithelial cell marker KLK3 was below detection in most tumours from castrated mice (62%, 23/37 mice), consistent with its known up-regulation by androgens. Endpoint tumour size after castration varied significantly in a PDX model-specific pattern; while most tumours were castration-sensitive (BM18, LuCaP70), the majority of LuCaP105 tumours continued to grow following castration. By contrast, LuCaP96 PDX showed a mixed response to castration. CTCs were detected in 33% of LuCaP105, 43% of BM18, 47% of LuCaP70, and 54% of LuCaP96 castrated mice using *RPL32* mRNA measurement in plasma. When present, CTC numbers estimated using human *RPL32* expression ranged from 1 to 458 CTCs per ml blood, similar to our previous observations in non-castrated mice. In contrast to their non-castrated counterparts, there was no relationship between tumour size and CTC burden in castrated mice. Unsupervised hierarchical clustering of the gene expression profiles of CTCs collected from castrated and non-castrated mice revealed distinct CTC sub-groups within the pooled population that were classified as having mesenchymal, epithelial, or EMP hybrid gene expression profiles. The epithelial signature was only found in CTCs from non-castrated mice. Hybrid and mesenchymal signatures were detected in CTCs from both castrated and non-castrated mice, with an emphasis towards mesenchymal phenotypes in castrated mice. Post-castration serum PSA levels were either below detection or very low for all the CTC positive samples highlighting the potential usefulness of CTCs for disease monitoring after androgen ablation therapy. In summary, our study of castration effects on prostate cancer PDX CTCs showed that CTCs were often detected in the castrate setting, even in mice with no palpable tumours, and demonstrated the superior ability of CTCs to reveal residual disease over the conventional clinical biomarker serum PSA.

## Introduction

Prostate cancer is the second most common cancer in men worldwide (Rawla, 2019). The disease can be characterised as localised, locally advanced or metastatic at diagnosis (Scher and Heller, 2000), and mortality is primarily attributed to metastasis. Therapy response monitoring is generally based on measurement of prostate specific antigen (PSA) in blood and imaging is used to monitor metastatic disease. Following advances in diagnostic capabilities, many patients are identified while the disease is still in its early stages, which has contributed to reductions in mortality. Despite this, prostate cancer is the fifth most common cause of cancer death globally and approximately 366,000 males die from prostate cancer annually (Pernar et al., 2018). As life expectancy has increased due to improvements in healthcare systems and incidence of prostate cancer increases with age, the probability of developing prostate cancer is also on the rise (Schatten, 2018).

Androgen deprivation therapy (ADT) is the most common non-surgical initial treatment for recurrent and treatment-naïve metastatic prostate cancer due to the universal and pivotal roles of androgens and androgen receptor (AR) in prostate growth and development and early stage prostate cancers (Shafi et al., 2013). In 1941, a relationship between androgens and prostate growth was established by Huggins and Hodges using animal model systems (Huggins et al., 1941; Toledo-Pereyra, 2001). AR is expressed on all luminal cells and occasionally on basal and stromal cells in normal prostate tissue (Shi et al., 2013; Cooper and Page, 2014). ADT encompasses surgical and chemical castration. Surgical castration involves orchiectomy through trans-scrotal surgery and is an irreversible process. Chemical castration can be effected by administration of oral or injectable estrogens, luteinizing hormone-releasing hormone (LH-RH) agonists, LH-RH antagonists, nonsteroidal antiandrogens or steroidal antiandrogens (Sharifi et al., 2005).

Despite good initial clinical responses to ADT (indicated by tumour regression and/or reduced serum PSA), most prostate cancers develop survival strategies that allow them to survive and proliferate in the presence of ADT (Scher and Heller, 2000). Clonal selection and evolution of tumour cells that can survive in the presence of castrate-levels of androgens, or by using AR-independent pathways, occurs. While androgen targeted therapies delay disease progression (Crawford et al., 2019), it is not curative and the patient ultimately succumbs to castrate-resistant prostate cancer (CRPC) (Scher and Heller, 2000). Disease progression can be identified by either an increase in serum PSA levels or clinical detection of new metastatic deposits (Cornford et al., 2021).

Cancer cells detected in patient blood provide a pool from which the initiators of distant metastasis are derived, as well as providing a snapshot of cells from existing tumour sites (both primary and metastatic). These cells are shed from tumours into the blood, where they are referred to as circulating tumour cells (CTCs). CTC analyses represents a minimally invasive liquid biopsy, in contrast to the current gold standard tumour biopsies for prostate cancer diagnosis. As CTCs are rare cells in the blood of most prostate cancer patients, sensitive detection technologies are required. CTC enumeration has been found to correlate well with progression free survival (PFS) and overall survival (OS) in prostate cancer patients (Armstrong et al., 2012; Diamond et al., 2012; Miyamoto et al., 2014; Pantel et al., 2019), and has been reported to outperform the traditional prostate cancer monitoring approach using serum PSA (De Bono et al., 2008). Furthermore, serum PSA levels do not always correlate with tumour progression and lack diagnostic specificity since blood levels of PSA can be elevated due to other benign prostate related diseases (Sadi, 2017). In addition, some types of prostate cancers, such as neuroendocrine and small cell cancers, do not produce PSA (Soundararajan et al., 2018; Patel et al., 2019).

Epithelial mesenchymal plasticity (EMP) of cancer cells - the ability to transition between epithelial and mesenchymal states - is a major pathway in malignant progression (Williams et al., 2019). In prostate cancer, EMP has been linked to castration resistance, alterations in androgen signalling and PSA expression (Nakazawa and Kyprianou, 2017), in addition to other manifestations of increased aggressiveness such as therapy resistance, migration, invasion and anoikis resistance (Micalizzi et al., 2017). EMP is particularly prevalent in CTCs (Bednarz-Knoll et al., 2012; Alix-Panabières et al., 2017). Controlling EMP signalling pathways may reduce the chances of developing resistance and metastatic spread, thus offering additional therapeutic avenues. The identification of additional treatment modalities for prostate cancer, and especially CRPC, is an active focus of research.

Recent studies have highlighted the importance of the E/M hybrid state (Pastushenko et al., 2018; Thompson and Nagaraj, 2018; Chakraborty et al., 2021; Deshmukh et al., 2021; Jolly et al., 2021). Hybrid CTCs, those cells that are simultaneously expressing epithelial and mesenchymal genes, have been shown to be a more aggressive population of cells with a higher metastatic potential than either fully epithelial or more mesenchymal states (Lecharpentier et al., 2011; Nieto et al., 2016; Basu et al., 2018; Denisov and Perelmuter, 2018; Pastushenko et al., 2018; Genna et al., 2020; Topel et al., 2020; Jolly et al., 2021). They also express stem cell markers and have self-renewal properties (Denisov and Perelmuter, 2018). Expanding our understanding of CTC molecular profiles present in prostate cancer, with a focus on EMP and the epithelial/mesenchymal hybrid state, may extend their clinical utility and open up new avenues for diagnosis and for the development of patient-specific precision medicine approaches.

Patient-derived xenograft (PDX) models recapitulate patient characteristics and allow us to more closely model disease than established cell lines that are grown in traditional 2-dimensional culture systems (Russell et al., 2017; Navone et al., 2018; Namekawa et al., 2019). Using a 42-gene human-specific RT-qPCR assay, we have previously detected CTCs with a hybrid EMP phenotype in breast (Tachtsidis et al., 2019) and prostate (Hassan et al., 2021a) cancer PDX models, and demonstrated significant changes in gene expression between tumours and their CTCs. A dysregulated EMP was observed with simultaneous increases in expression of both epithelial and mesenchymal gene expression in CTCs. CTCs were also found to be very heterogenous in enumeration, size and gene expression.

In the current study we have examined prostate PDX and PDX-CTC expression of EMP-associated genes in the context of castration to model ADT. Surprisingly, relatively high levels of CTCs were frequently detected after castration, even when the PDX tumours appeared fully regressed. We found that certain EMP related genes were associated with castration-resistance in the tumours of PDX models, as well as differences in the EMP prolife of CTCs collected from castrated mice compared to CTCs of non-castrated mice.

## Methodology

### Mouse Blood and Tissue Collection

Four prostate cancer PDX models (BM18, LuCaP70, LuCaP96 and LuCaP105) (McCulloch et al., 2005; Nguyen et al., 2017; Hassan et al., 2021b) were grown continuously as subcutaneous tumours in the lateral flank of male severe combined immune-deficient (SCID) mice. At passage, a tumour chunk (approximately 2 mm^3^) was implanted subcutaneously into each mouse. Once tumours reached 100–600 mm^3^ in volume, bilateral orchioepididymectomy was performed under isoflurane anaesthesia to surgically castrate the mice. Mice were killed at variable days post castration based on tumour volume (BM18 92 ± 5, LuCaP70 88 ± 24, LuCaP96 89 ± 33, LuCaP105 67 ± 28 days post-castration). Samples were collected sequentially at routine passaging over a period of 2.5 years. PDX work was carried out in accordance with Australian National Health and Medical Research Council (NHMRC) guidelines and under approval by the relevant Animal Research Ethics Committees (TRI/QUT/370/17, QUT1800000289). The workflow and sample numbers are summarised in Supplementary Figure S1.

On the day of castration, a blood sample was collected from the submandibular vein for PSA analysis. At endpoint, 0.8–1.0 ml of blood was collected from each mouse via cardiac puncture and split into two samples for PSA and CTC analyses.

For PSA analysis, blood was added to a 1.5 ml microfuge tube and allowed to clot at room temperature. Serum was collected and stored at −80°C prior to PSA measurement.

For CTC analyses, blood from the terminal bleed (0.2–0.5 ml of blood) was added to a 1.5 ml microfuge tubes containing 25 µl of 2.5 M EDTA.

Pieces of tumour were also collected where material was available (tumours in some mice regressed fully in response to castration and thus no tissue was available from these mice), and a portion immediately placed into RNAlater™ Stabilization Solution (Thermo Fisher Scientific, Waltham, MA) and stored overnight prior to being stored at −80°C until RNA extraction.Where samples from non-castrated PDX-bearing mice are compared to castrated mice, the data for non-castrated mice has been recently published (Hassan et al., 2021b).

### Sample Processing

Blood samples for CTC analyses were processed within 3 h of collection. RBC lysis buffer (2 ml; G-Biosciences, St. Louis, MO) was added to each sample and incubated for 5 min prior to centrifugation at 400 × *g* for 10 min at room temperature to obtain cell pellets for RNA analysis or immunocytochemistry (ICC).

For RNA analyses, the supernatant was removed and RNA lysis buffer from ISOLATE II RNA Mini Kit (Bioline^©^, Australia) added to the cell pellets and briefly vortexed before storing at −80°. RNA was subsequently extracted using the same ISOLATE II RNA Mini kit, following the manufacturer’s protocol.

For ICC, the cell pellets were resuspended in 600 µl Dulbecco’s modified Eagle’s medium (DMEM) containing 10% fetal bovine serum (FBS; Gibco, Thermo Fisher Scientific), and antibiotics penicillin and streptomycin (Gibco). Each sample was split onto three slides (200 µl per slide) and cytospun at 1,000 g for 5 min with medium acceleration. The samples were then stored at −80°C until staining was performed as below.

Tumours were homogenised using a TissueLyser II (Qiagen, Germany) and 5 mm diameter stainless steel beads. To avoid RNA degradation, RNA Lysis buffer from the ISOLATE II RNA Mini Kit was added to the tumour pieces prior to homogenisation. RNA was extracted using the same kit, following the manufacturer’s protocol.

### Immunocytochemistry (ICC) Analysis

Cytospun cells were fixed using 4% neutral buffered formalin (Sigma-Aldrich, Missouri, MO) following by permeabilization with 0.4% Triton X-100. For background blocking, cells were incubated for 15 min with Background Sniper (Biocare Medical, Pacheco, CA) followed by 1 h with 5% bovine serum albumin (BSA; Sigma-Aldrich). The samples were incubated with anti-vimentin human-specific primary antibody (V9; cat no. 790-2917; Ventana, AZ) and an antibody cocktail against cytokeratins 8, 18 and 19 (anti-KRT8 (HPA049866), anti-KRT18 (HPA001605), anti-KRT19 (HPA002465); Sigma-Aldrich) overnight, followed by secondary antibodies labelled with Alexa Fluor™ 647 and Alexa Fluor™ 488 (Invitrogen, Waltham, MA), respectively, for 2 h. The slides were covered in mounting solution mowiol and coverslips added. Slides were scanned using VS200 Olympus Slidescanner at ×20 magnification (40 × 0.27 µm resolution), and images collected and assessed using OlyVIA V3.3 (Olympus, Japan). Total CTCs were determined from the combined sum of all stained cells.

### Molecular RT-qPCR Analysis

RT-qPCR was performed as described previously, using custom-designed human-specific primers (Tachtsidis et al., 2019; Hassan et al., 2021b). Briefly, cDNA was synthesised using SuperScript™ IV First-Strand Synthesis System (Invitrogen) on a GeneAmp™ PCR system 9700 (Applied Biosystems, Waltham, MA) followed by cDNA precipitation. Samples were then pre-amplified using 2x SYBR premix for 15 cycles followed by a 1:125 dilution. 2x SYBR premix was then used to perform qPCR using a Life Technologies ViiA™ 7 Real-Time PCR System (Thermo Fisher Scientific) in 384 well plate format.

### Estimation of Total CTC Number per ml of Mouse Blood

A positive control sample (PDX LuCaP141 tumour piece) was used to estimate the number of CTCs. Each mammalian cell contains an estimated 10–30 pg of RNA (Sambrook and Russell, 2000). We used 10 ng RNA for our CTC estimation, which is ∼1,000 cell equivalent of RNA in the RT-qPCR protocol. The *RPL32* housekeeper gene Ct value was used to estimate the number of CTCs, assuming that each cycle represents a 2-fold change in signal. The values were adjusted based on the volume of sample used (25%) and expressed as estimated number of CTCs per ml of blood.

### Enzyme-Linked Immunosorbent Assay (ELISA) for PSA

ELISA was performed on serum samples from pre-castration and post-castration mice using the Genway Total PSA ELISA Kit (San Diego, CA) following the manufacturer’s protocol. Owing to expected high PSA concentrations of pre-castration serum samples based on previous measurements in serum from mice bearing these PDXs, these samples were diluted 1:3 prior to performing the assay. Post-castration samples were used undiluted, and the assay repeated with diluted serum if results exceeded the upper limits of the assay. Test samples (25 µl) were added to individual wells in the plate and covered in 100 µl conjugate solution. After a 30 min incubation in the dark at room temperature, the solution was aspirated and wells were washed 3 times with 300 µl diluted wash solution. After this, 100 μl TMB Substrate Solution was added to each well and the plate incubated for 15 min in the dark. Lastly, 100 µl Stop Solution was added to each well and the plate was imaged using a FLUOstar^®^ Omega microplate reader (BMG Labtech, Germany) at an absorbance of 450 nm.

### Statistical Analysis

All statistical work was performed using Microsoft^®^ Excel (version 2110), GraphPad Prism 8.3.0 and Morpheus (https://software.broadinstitute.org/morpheus/). Correlation analysis was performed using non-parametric Spearman’s correlation and a 95% confidence interval. Unpaired two-tailed *t* test was performed with *p* value less than 0.05 as significant and false discovery was determined using Two-stage linear step-up procedure of Benjamini, Krieger and Yekutieli, with Q = 5%.

## Results

### Monitoring Serum PSA Levels of PDX-Bearing Mice

**FIGURE 1:**
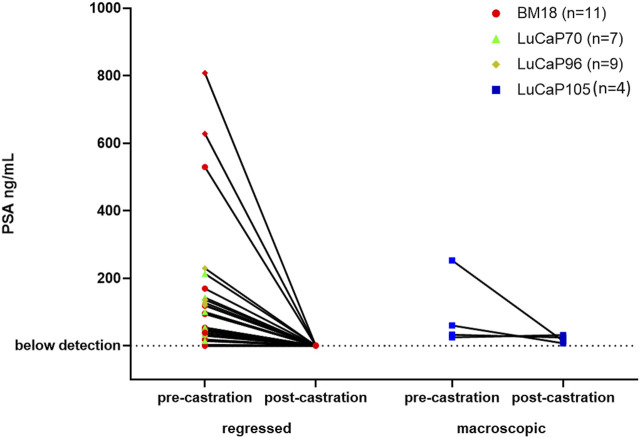
Serum PSA levels of mice pre- and post- castration. PSA levels after castration were very low or not detected in mice with regressed tumours. Post-castration PSA levels were significantly higher in mice where no tumour regression was observed compared to mice with regressed tumours (unpaired *t*-test, *p* = 0.0007). Tumours that decreased in size from their pre-castration measurement and weighed less than 0.3 g were classified as regressed.

Serum PSA levels are routinely used to monitor prostate cancer disease progression. We tested PSA levels in serum samples collected from mice immediately prior to castration and following castration at endpoint (Supplementary Figure S1). To identify whether the serum PSA levels correlated with castration resistance, we compared them in mice with regressed and non-regressed tumours (Figure 1). Post-castration, serum PSA levels were either non-detectable or extremely low for mice with regressed tumours. For mice that had growing tumours following castration, low post-castration serum PSA levels were detected in all samples (*n* = 4), with 1 of these samples, LuCaP105 (tumour weight = 0.52 g), having slightly increased serum PSA post-castration.

### CTC Identification Using Immunofluorescence Analysis

**FIGURE 2:**
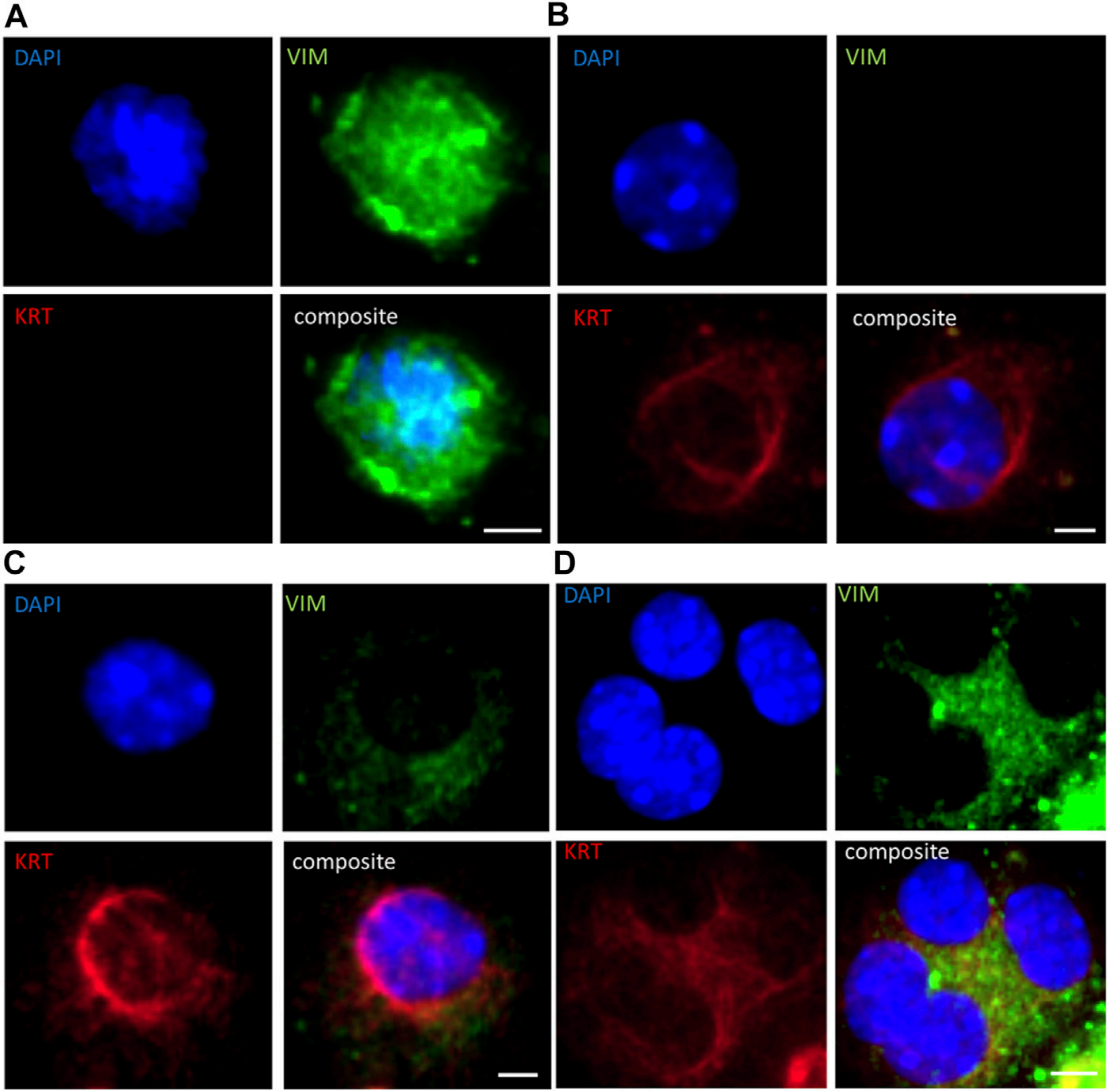
Immunofluorescence staining for **(A)** vimentin (VIM; green) only, **(B)** epithelial cytokeratins (KRT; cytokeratins 8/18/19; red) only, **(C)** hybrid cells (both VIM and KRT; orange), and **(D)** cluster of hybrid cells in PDX-derived CTCs in a representative blood sample from a castrated LuCaP96 xenograft mouse. Scale bar denotes 5 µm.

All BM18 (*n* = 34), 20/21 LuCaP70, 21/29 LuCaP96 and 5/17 LuCaP015 tumours showed regression (Supplementary Figures S2A–D). Blood samples were collected from all castrated mice. Of these bloods, 13 randomly selected samples (4 BM18, 4 LuCaP70, 3 LuCaP96 and 2 LuCaP105) were examined using ICC for presence of cells expressing mesenchymal vimentin (VIM) and/or epithelial cytokeratins 8/18/19 (KRT) (Figure 2; Table 1). A negative control blood sample from a mouse without a tumour was also examined and no VIM^+^ or KRT^+^ cells were identified (Supplementary Figure S3). In PDX-bearing mice, the total number of CTCs observed varied between 22 and 2,725 cells per ml of blood, and no significant difference in CTC numbers was seen between castrated and non-castrated mice (BM18 (*p* = 0.387), LuCaP70 (*p* = 0.867), LuCaP96 (*p* = 0.256), LuCaP105 (*p* = 0.295)). Hybrid CTCs expressing both VIM and KRT were the most commonly identified type of CTC observed in 7/13 castrated samples, followed by epithelial CTCs (5/13).

**TABLE 1:** Quantification of cytokeratin/vimentin (KRT/VIM) immunofluorescence staining of castrated PDX blood samples.

PDX ID	Total CTCs (CTCs/ml)	KRT^+^VIM^−^ (%)	KRT^−^VIM^+^ (%)	KRT^+^VIM^+^ (%)
BM18-133-R	1,483	32	47	21
BM18-143-R	168	18	8	74
BM18-149-R	410	2	37	61
BM18-121-R	22	18	36	45
LuCaP70-111-R	2,186	69	0	31
LuCaP70-139-R	154	58	21	21
LuCaP70-106-R	74	8	19	73
LuCaP70-158-R	28	79	7	14
LuCaP96-116-R	2,725	62	4	34
LuCaP96-159-R	255	16	13	72
LuCaP96-148-R	363	9	13	78
LuCaP105-134-G	170	66	8	26
LuCaP105-101-R	988	3	38	59

-G: growing tumour; -R: regressed tumour.

CTC clusters were observed in all blood samples (Figure 2; Table 2). The number of CTCs varied between 2 and 77 CTCs per cluster, and up to 330 clusters per ml of blood (LuCaP96). CTC phenotype in individual clusters was always homogenous, with each cluster containing either KRT^+^ only, VIM^+^ only, or hybrid (KRT^+^/VIM^+^) cells. CTC clusters were rarely comprised of cells only expressing VIM, which were detected in only 5/13 blood samples.

**TABLE 2:** CTC cluster counts in castrated PDX blood samples using cytokeratin/vimentin (KRT/VIM) immunofluorescence staining.

PDX ID	Total Clusters (Clusters/ml)	KRT^+^VIM^−^ (%)	KRT^−^VIM^+^ (%)	KRT^+^VIM^+^ (%)
BM18-133-R	27	37.5	25	37.5
BM18-143-R	6	0	0	100
BM18-149-R	18	0	29	71
BM18-121-R	4	50	0	50
LuCaP70-111-R	160	22.5	0	77.5
LuCaP70-139-R	6	33	0	67
LuCaP70-106-R	14	0	0	100
LuCaP70-158-R	4	100	0	0
LuCaP96-116-R	330	54.5	1.5	44
LuCaP96-159-R	23	11	0	89
LuCaP96-148-R	60	5.5	5.5	89
LuCaP105-134-G	18	78	0	22
LuCaP105-101-R	40	15	15	70

### RT-qPCR Pre-Screening of Blood Samples for the Presence of CTCs

**FIGURE 3:**
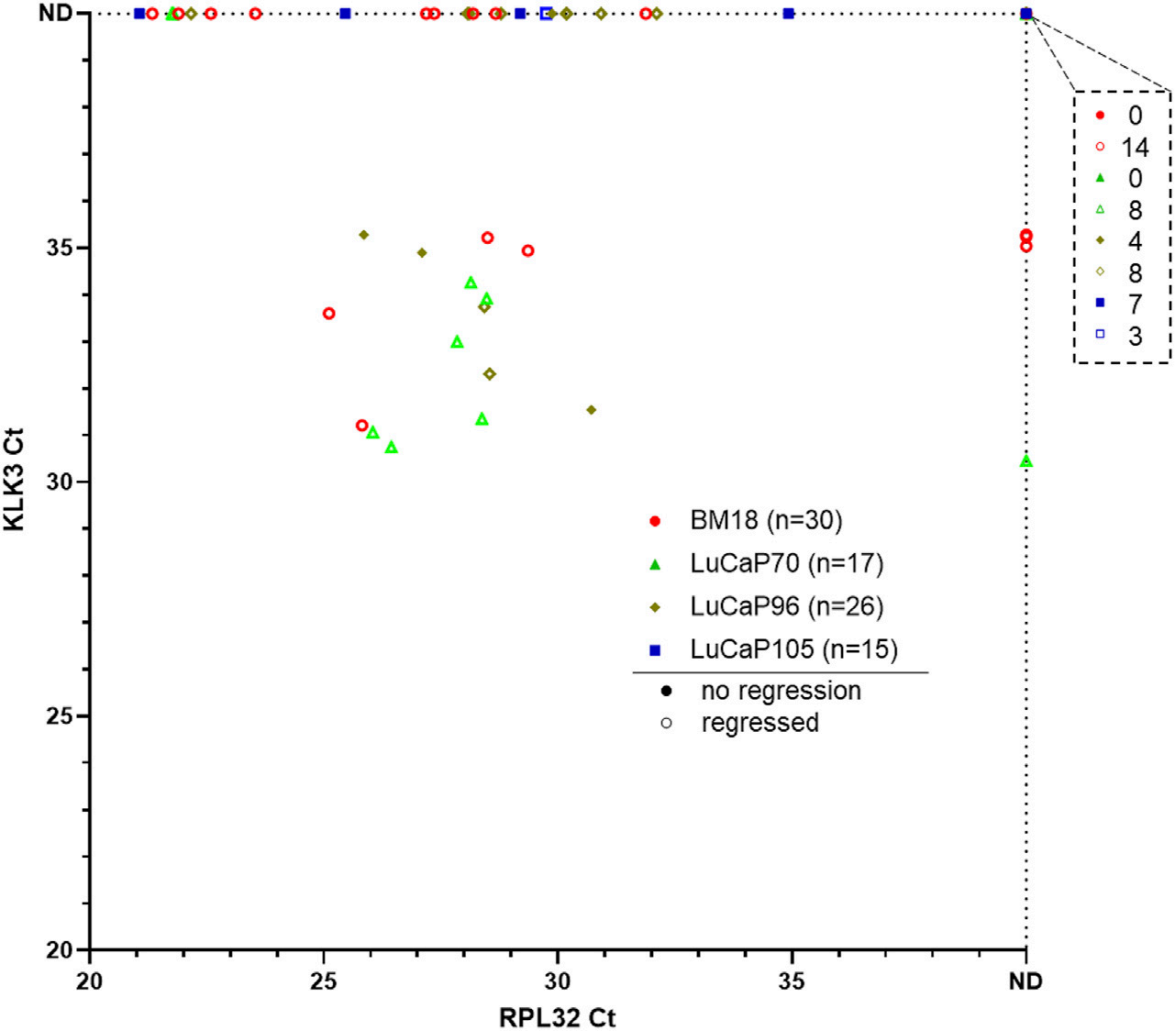
Gene expression results for pre-screen of blood collected from prostate cancer PDX-bearing castrated mice. Raw Ct values for RPL32 and KLK3 genes from four PDX models are plotted, with smaller values representing higher gene expression and each symbol corresponding to a blood sample. 13/30 BM18, 8/17 LuCaP70, 14/26 LuCaP96 and 5/15 LuCaP105 were RPL32 positive. Four samples showed expression of KLK3 in the absence of RPL32. The number of samples that were negative for both RPL32 and KLK3 is indicated in the top right breakout box. Each point denotes a sample and hollow points represent samples from mice with regressed tumours. ND, not detected.

The remainder of the blood samples (*n* = 88) from 30 BM18, 17 LuCaP70, 26 LuCaP96 and 15 LuCaP105 castrated PDX-bearing mice were used for RNA-based analysis. A pre-screen was performed on all blood samples to identify CTC-positive samples using human-specific *RPL32* and *KLK3* primers (Figure 3). We have previously confirmed specificity of this assay using a tumour-free mouse (Hassan et al., 2021b). From 88 blood samples, 42 were *RPL32*-positive (BM18:13/30; LuCaP70: 8/17; LuCaP96: 14/26; LuCaP105: 5/15), of which 17 also had measurable *KLK3* expression (BM18: 4/15; LuCaP70: 7/8; LuCaP96: 5/14; LuCaP105: 0/5). *KLK3* was often below detection, as expected in castrated mice because *KLK3* expression is positively regulated by androgens. Four samples showed expression of *KLK3* in the absence of *RPL32* (3 BM18 and 1 LuCaP70), however only samples that were positive for *RPL32* were used for further analysis (as per predefined criteria (Hassan et al., 2021b)).

### Human-Specific RT-qPCR Profiling of Blood Samples from Castrated PDX-Bearing Mice

**FIGURE 4:**
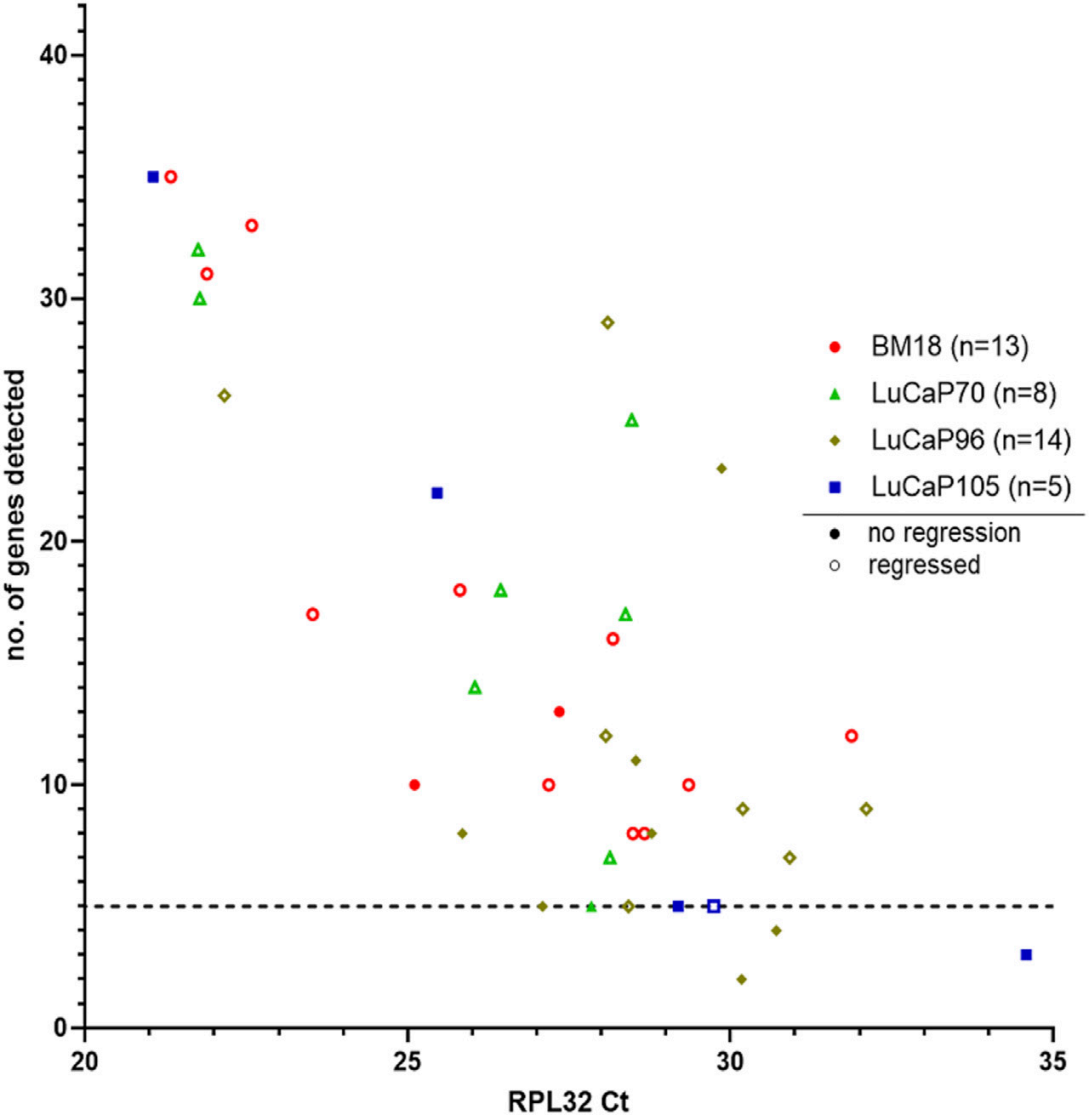
Human-specific RT-qPCR analysis of PDX-bearing mouse blood samples. Scatter plot for RPL32 raw Ct value against number of genes detected (maximum = 42), where each symbol corresponds to an individual mouse. Low Ct values correspond to higher gene expression. A correlation was observed between number of genes detected and RPL32 expression (*p* = 0.0000075, r = −0.63). Samples that had less than five genes detected (1 LuCaP105 and 2 LuCaP96) were excluded from further analysis.

A 42-gene human-specific tandem-nested RT-qPCR assay was used in this study as previously described (Tachtsidis et al., 2019; Hassan et al., 2021b). This comprised of epithelial-associated genes (*JUP, KRT20, KLK3, CDH1, GRHL2, EPCAM, BMP7, CLDN3, CLDN4*, and *CLDN7*), mesenchymal-associated genes (*SNAI1, VIM, NOTCH1, EGFR, FN1, SERPINE1, SNAI2, VCL, IGF1R, RRAS, FOSL1, MSN, NRP1, LAMC2, TNC, EMP3* and *INHBA*), hypoxia-associated genes (*APLN, HIF1A*, and *BNIP3*), cancer stem cell (CSC) markers (*CD24* and *CD44*), hormonal regulation (HR) genes (*ESR1, PGR*, and *TFF1*), selected other genes (*PPARGC1A, ILK*), and housekeeper genes (HKGs; *RPL32, GUSB, TBP, OAZ1* and *NONO*). The complete 42 gene RT-qPCR was run on all *RPL32* positive blood samples (*n* = 40). The number of genes detected per sample varied and showed a positive correlation with the level of *RPL32* gene expression (Figure 4; *p* = 0.0000075, r = −0.63). In order to analyse samples that had sufficient data points to provide meaningful results, only samples with detectable expression of at least five genes were included in further analysis (BM18, 13; LuCaP70, 8; LuCaP96, 12; LuCaP105, 4) (Figure 4; Table 3).

**TABLE 3:** Summary table for number of samples positive by RT-qPCR for each PDX model.

PDX ID	Total samples (*n*)	Positive samples^a^ (*n*)		Percentage positive (%)	Percentage positive for non-castrated mice (%)
		Regressed	Non-regressed		
BM18	30	13	0	43	50
LuCaP70	17	7	1	47	69
LuCaP96	26	8	4	46	77
LuCaP105	15	1	3	27	50

Regressed: tumour decreased in size following castration and weighed less than 0.3 g at endpoint analysis.

a Samples with at least five genes detected were considered positive.

**FIGURE 5:**
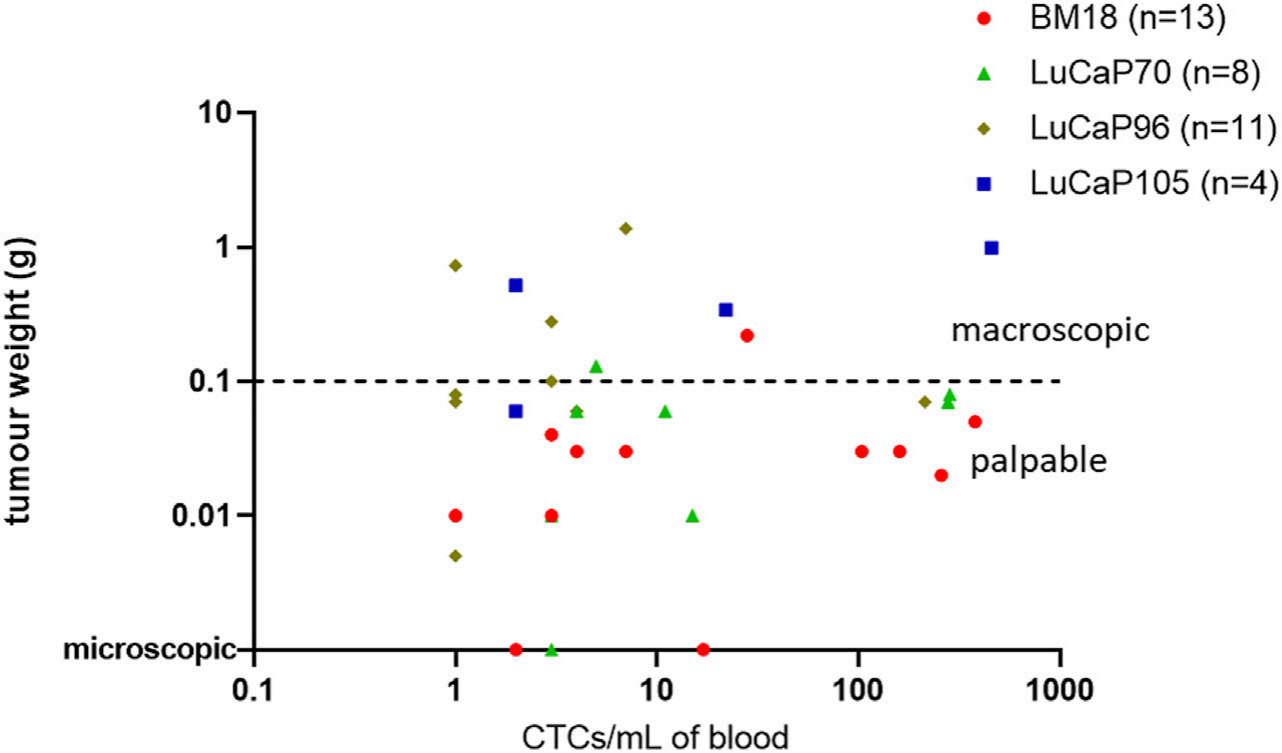
Scatter plot comparing endpoint tumour weight (g) of PDX-bearing castrated mice to the estimated number of CTCs per mL in blood (based on mRNA levels of RPL32). Each point denotes an individual mouse. Most tumours responded to castration with regression, including three mice having only microscopic tumours at endpoint (*n* = 27/37). The estimated number of CTCs was not significantly related to tumour weight using nonparametric Spearman correlation and a 95% confidence interval (*p* = 0.50, r = 0.12). Note: One sample with unknown tumour weight at endpoint (LuCaP96) was omitted from this graph.

The endpoint tumour sizes varied significantly. The majority of PDXs responded to castration, decreasing in size and regressing to <0.3 g at endpoint. Most of these mice had only just palpable (but not measurable using callipers) or microscopic tumours at the time of endpoint analysis. By contrast, 16% of primary tumours–mainly LuCaP105 and some LuCaP96 - continued to grow following castration (castration-resistant). The number of estimated CTCs (based on *RPL32* mRNA levels) ranged from as few as 1 to as many as 458 CTCs per ml of blood (mean = 66, median = 4). No correlation was observed between endpoint tumour weight and the estimated number of CTCs per ml of blood (*p* = 0.50, r = −0.12; Figure 5). In fact, most PDXs with detectable CTCs had no palpable tumour (24/37). While most BM18 samples had low to moderate estimated numbers of CTCs (mode = 3), four samples had strikingly high numbers of estimated CTCs (*n* = 104, 160, 258, 380). Interestingly, all of these samples had only just palpable or microscopic tumours, consistent with the robust response to castration always demonstrated by BM18 PDXs (Supplementary Figure S2). An additional 3 PDX-bearing mice had high numbers of CTCs despite no palpable tumours being present at endpoint (two LuCaP70 (*n* = 284, 279), one LuCaP96 (*n* = 214)). Furthermore, one LuCaP105 PDX also had a high number of CTCs (*n* = 458) in the context of a large tumour (tumour weight = 0.99).

We compared our previous estimates of CTCs in non-castrated mice (Hassan et al., 2021b) with those reported here in castrated mice. No significant difference in the number of CTCs for castrated and non-castrated mice was observed in BM18 (*p* = 0.235), LuCaP70 (*p* = 0.186), LuCaP96 (*p* = 0.289) or LuCaP105 (*p* = 0.927) PDX models. We also compared post-castration serum PSA with CTC enumeration and found no significant correlation (*p* = 0.191, r = 0.271) (Supplementary Figure S4).

**FIGURE 6:**
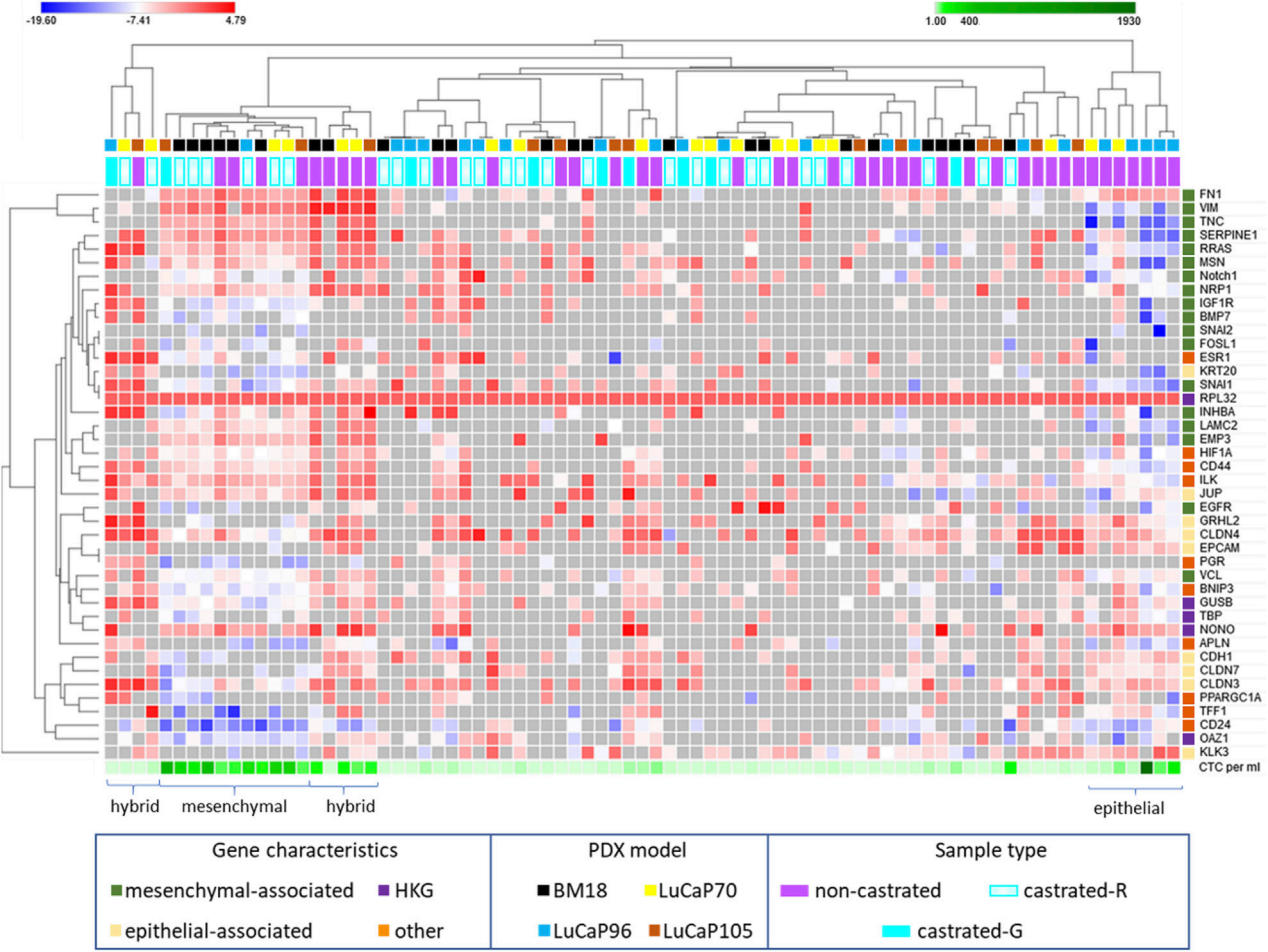
Heatmap of RPL32 normalised ΔCt values for 42 genes following RT-qPCR analysis of blood samples from castrated and non-castrated PDX-bearing mice. The data for non-castrated mice was reported previously (Hassan et al., 2021b). Hierarchical unsupervised clustering was performed using one minus Pearson correlation and global normalisation. Bright red denotes high expression and bright blue denotes low expression of individual genes. Grey represents no detection. Each row corresponds to a gene and each column corresponds to a blood sample. Four clusters, epithelial, hybrid (x2), and mesenchymal associated, are indicated. Whether castrated mice had regressed (castrated-R) or growing (castrated-G) tumours is indicated.

The complete 42 gene RT-qPCR panel assessment for all CTC positive blood samples from castrated mice allowed assessment of EMP-related changes (Figure 6). Unsupervised hierarchical clustering of these blood samples with blood samples from non-castrated mice bearing the same PDX models revealed four main clusters (one epithelial, two hybrid, one mesenchymal). The remainder of the samples had generally relatively low gene expression and CTC burden.

One tight cluster (mesenchymal) of 11 samples, predominantly comprising of BM18 samples, had a strongly mesenchymal-dominated gene expression signature. Several mesenchymal-associated genes had uniformly high expression in this cluster, including *FN1, VIM, TNC, SERPINE1, MSN, RRAS, NRP1, LAMC2, INHBA* and *EMP3*, as had one epithelial-associated gene *JUP*, and other genes including *HIF1A* (hypoxia), *NONO* (housekeeper gene), *CD44* (CSC) and *ILK* (anoikis). While *CD44* was uniformly high in all these samples, *CD24* was uniformly low, a common CSC/mesenchymal gene expression pattern (Mani et al., 2008). The epithelial marker *EPCAM* was only detected in one of these samples, in which its expression was low, and all other epithelial-associated genes except *JUP* had low or no expression. Expression of the epithelial-associated *KLK3* gene was undetectable in 9/11 samples, with low expression in the remaining two samples. Interestingly, all the castrated mouse bloods in this cluster were from mice with regressed tumour at endpoint, except for a single LuCaP105 sample. This LuCaP105 PDX bearing mouse had the largest tumour at endpoint (0.99 g) and the highest number of CTCs in our castrated mouse cohort (458 CTCs per ml of blood). CTC enumeration was uniformly high for all samples in this cluster (mean = 211, median = 214). Only 3/11 samples in this cluster were from non-castrated mice (2x BM18, 1x LuCaP105).

Two distinct EMP hybrid gene expression clusters appeared to have very similar gene expression patterns but did not cluster alongside each other on the heat map. One of the two hybrid-like clusters had samples with high numbers of CTCs (mean = 67, median = 82), while the other had consistently low CTCs numbers (*n* = 1, 1, 3, 11), which may underpin their separation. In both hybrid clusters, a relatively large number of epithelial (*n* = 9) and mesenchymal genes (*n* = 12) had strikingly high expression. Interestingly, genes were either expressed very highly or they were below detection in this cluster. Like the mesenchymal cluster, these hybrid clusters also had a stem cell like phenotype with high expression of *CD44* and low expression of *CD24*.

The third cluster (epithelial) only contained blood samples from non-castrated mice, and was comprised of only LuCaP96 (*n* = 5) and LuCaP70 (*n* = 2) samples. This cluster had a relatively epithelial phenotype with high levels of expression of epithelial-associated genes *CDH1, GRHL2, CLDN3, CLDN4, CLDN7, EPCAM* and *KLK3* and only high expression of one mesenchymal-associated gene (*FN1*). Low levels of both *CD24* and *CD44* gene expression were detected, indicating an absence of CSC phenotype. CTC enumeration for this cluster was high (214–1927 CTCs per ml of blood).

Other samples in the experiment had insufficient numbers of detectable gene expression to determine their EMP state.

**FIGURE 7:**
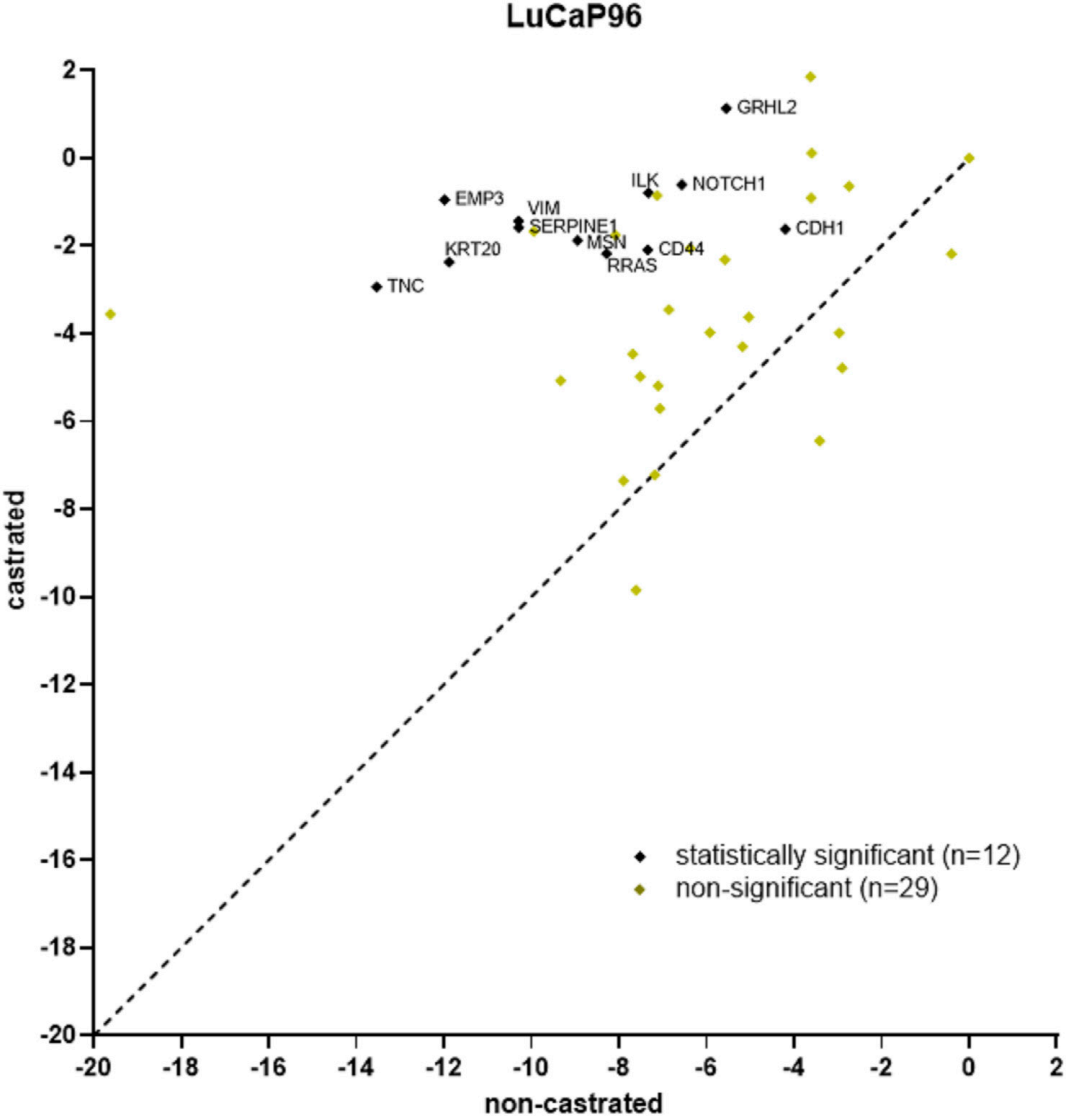
Scatter plot of LuCaP96 model CTC gene expression RT-qPCR results for castrated versus non-castrated (data from (Hassan et al., 2021b)) mice. 12/41 genes had significantly different expression (*p* < 0.05). RPL32 normalised ΔCt values are plotted and each point denotes an individual gene. Significant differences in gene expression values between CTCs from castrated and non-castrated mice were identified using the Benjamini, Krieger and Yekutieli two-stage linear step-up procedure to control the false discovery rate (Q = 5%, *p* < 0.05).

The least gene expression variation between tumours and their CTCs was previously observed in non-castrated LuCaP96 PDX mice (Hassan et al., 2021b). After castration, LuCaP96 samples dominated the epithelial cluster and were rarely in the mesenchymal (*n* = 1/11) or hybrid (*n* = 1/9) clusters (Figure 6). We therefore delved deeper to understand the unique findings in this model. In the LuCaP96 model, 12/41 genes had significantly different expression between castrated and non-castrated mice blood samples (*VIM, NOTCH1, SERPINE1, RRAS, MSN, TNC, KRT20, CDH1, CD44* and *ILK*) based on a two-tailed unpaired *t*-test (*p* < 0.05; Figure 7). Other PDXs showed no significant difference in gene expression (Supplementary Figures S5A–C). Overall, no significant differences between castrated and non-castrated mouse blood samples were observed when all PDXs were analysed together (Supplementary Figure S5D).

**FIGURE 8:**
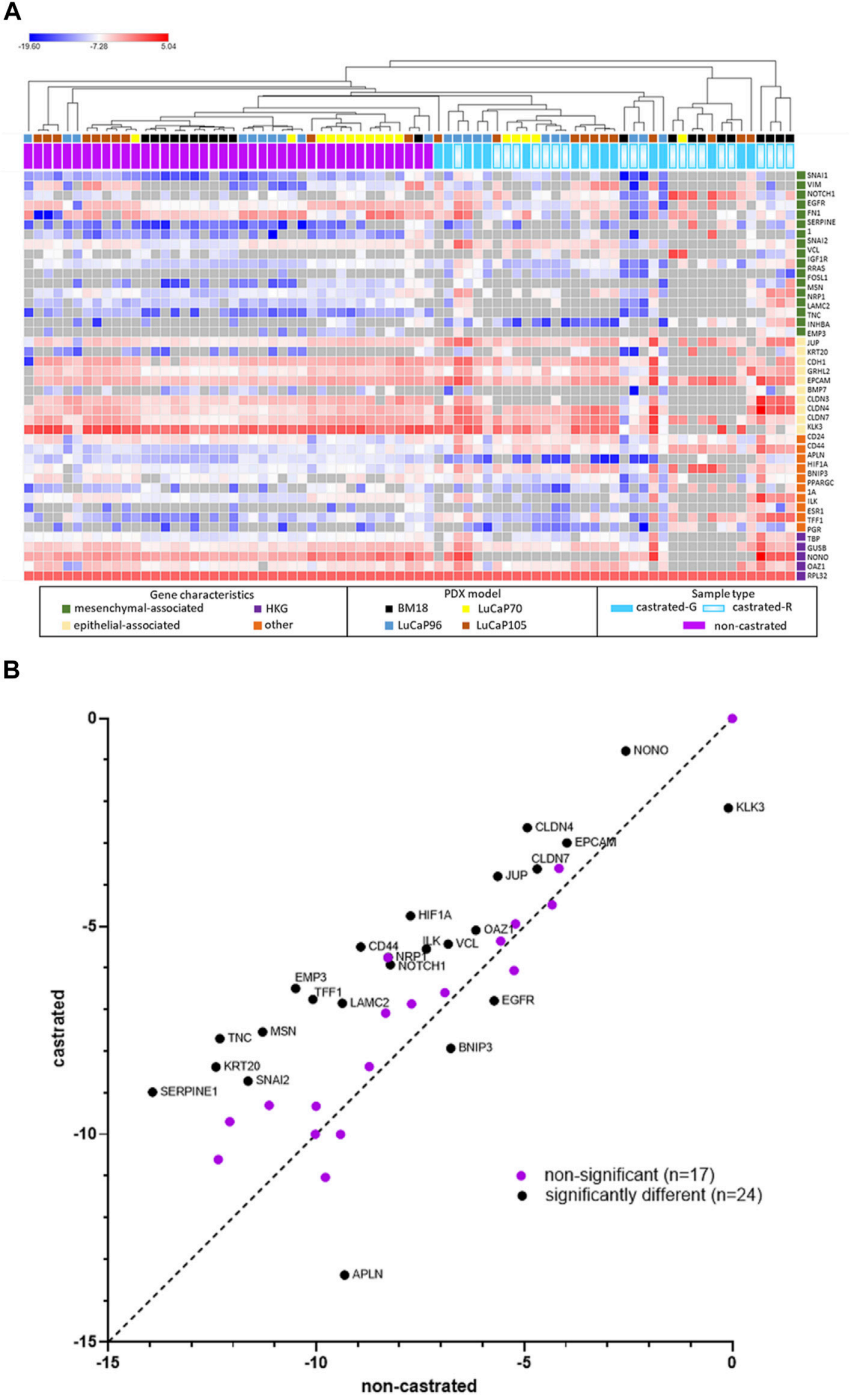
RPL32 normalised ΔCt values for 42 genes using RT-qPCR analysis of tumour samples from castrated and non-castrated PDX-bearing mice. The data for the non-castrated mice has been published previously (Hassan et al., 2021b). **(A)** Heat map represents individual samples, where each row corresponds to a gene and each column corresponds to a tumour sample. Unsupervised hierarchical clustering was performed using one minus Pearson correlation and global normalisation. Bright red denotes high expression and bright blue denotes low expression of individual genes. Grey represents no detection. Tumour status in castrated mice is indicated (regressed (castrated-R) or growing (castrated-G)). **(B)** Scatter plots of overall tumour gene expression results for non-castrated PDX mice versus castrated mice. Each point denotes an individual gene. Unpaired, two-tailed *t*-tests showed 24 genes had significant difference for gene expression (*p* < 0.05).

Tumour samples from castrated mice (BM18, 13; LuCaP7, 6; LuCaP96, 11; LuCaP105, 12), were assessed using the RT-qPCR assay panel and compared with tumours from non-castrated mice (Figure 8A). The majority of BM18 and LuCaP70 tumours were castration sensitive (regressed in response to castration), whereas LuCaP105 tumours were mostly castrate-resistant. By contrast, the LuCaP96 model showed a variable response to castration and therefore samples representing both castrate-sensitive (*n* = 6) and castrate-resistant (*n* = 5) tumours were assessed. Overall, the tumours had epithelial-like gene expression regardless of PDX, showing high expression of most epithelial-associated genes and low expression of most mesenchymal-associated genes. Upon unsupervised hierarchical clustering, tumours from castrated and non-castrated mice were clearly separated into two groups (castrated and non-castrated). Some of the tumours from castrated mice (*n* = 19) clustered closer to those from non-castrated mice than the remaining tumours from castrated mice. Interestingly only 12/19 (50%) of these tumours had regressed, compared to 10/13 (77%) of the remaining tumours.

Overall, there were significant differences in gene expression between tumours from castrated and non-castrated mice for 24 genes (Figure 8B). Some of these differences were also seen in individual models (Supplementary Figures S6A–D), while others were only seen in the combined analysis. LuCaP96 had the fewest significantly different genes (*APLN* and *CD44*). The samples were further analysed into separate groups of castrate-resistant and castrate-sensitive tumours, however, the number samples is small and consequently the number differently expressed genes was still limited (castrate-resistant = 2/41 genes, castrate-sensitive = 5/41 genes) (Supplementary Figures S7A,B).

All blood and tumour samples from castrated and non-castrated mice were collectively analysed irrespective of PDX type (Supplementary Figures S8A–D). For the purpose of this analysis, samples from castrate-sensitive and castrate-resistant mice were addressed separately. Epithelial-associated genes *KRT20* and *BMP7* were consistenantly upregulated in all blood samples as compared to tumours. Mesenchymal-associated genes *VIM, SERPINE1, RRAS, FOSL1, MSN, INHBA* and *EMP3* were also upregulated in blood samples.

There was significant difference in gene expression between tumours and blood samples for a number of genes from non-castrated mice for BM18, LuCaP70 and LuCaP105 models (Hassan et al., 2021b), while only three genes were significantly different for LuCaP96 (Table 4). Interestingly, the reverse trend was observed in terms of castrated mice, where only LuCaP96 had a large number of differentially expressed genes.

**TABLE 4:** Summary of gene expression differences between CTC and primary tumour samples from castrated and non-castrated mice across PDX models.

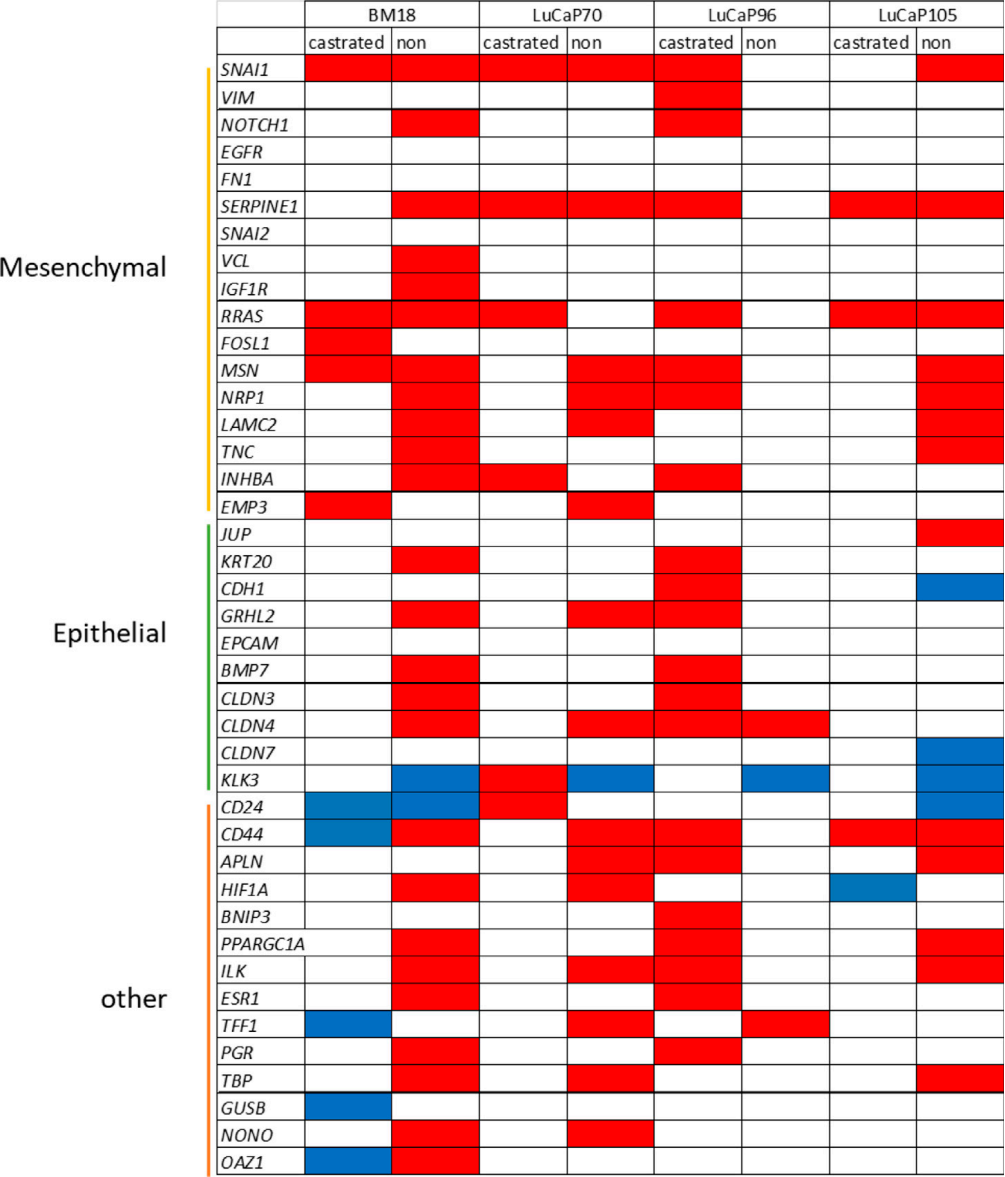

Red boxes depict upregulation (higher in CTCs compared to primary tumour); blue boxes depict downregulation (lower in CTCs compared to primary tumour); white boxes have either no significant change (*p* > 0.05) or insufficient data for analysis.

Significant differences in gene expression values between CTCs and tumours were identified using unpaired unsupervised *t*-test.

## Discussion

Androgens play a pivotal role in the growth and development of normal prostate gland as well as prostate tumour cells (Wilson, 2011; Shafi et al., 2013), and ADT is the initial non-surgical treatment for almost all prostate cancer patients. Despite initial response to therapy, many prostate cancer patients develop castrate-resistant metastatic disease. The molecular basis underpinning the development of metastatic CRPC is thus an active area of research (Amaral et al., 2012; Shafi et al., 2013; De Nunzio et al., 2017; Vlachostergios et al., 2017; Davies et al., 2019). Androgens provide a key differentiation signal for prostate epithelial cells, and androgen deprivation has been shown to lead to EMP induction (Sun et al., 2012; Nouri et al., 2014; Bishop et al., 2015; Nakazawa and Kyprianou, 2017; Tiwari et al., 2020). Thus EMP likely contributes to this disease progression, however its role is not yet fully understood (Børretzen et al., 2019; Laudato et al., 2019), and some contradictions exist. Zhu and Kyprianou (Zhu and Kyprianou, 2010) showed that androgens and AR signalling can result in an EMT induction, although this only occurred in the context of low AR levels and observations were limited to LNCaP and PC-3 prostate cancer cell lines. By contrast most other studies demonstrate that androgen deprivation promotes EMT (Sun et al., 2012; Nouri et al., 2014; Bishop et al., 2015; Tiwari et al., 2020). Numerous studies have shown that ADT can result in an increase in expression of mesenchymal markers such as vimentin, N-cadherin and TWIST1, and a decrease in epithelial markers, especially E-cadherin, in prostate cancer PDX model and patient tumours (Nouri et al., 2014). In the LuCaP35 PDX model, castration led to EMT in the tumours, a change in phenotype that was characterised by a decrease in E-cadherin coupled with an increase in N-cadherin and vimentin protein levels (Sun et al., 2012). In the same study, castration also induced these changes in benign prostate epithelial cells in non-PDX-bearing mice (Sun et al., 2012). A mesenchymal to epithelial reverting transition signature specific to reversal of EMT was enriched in metastatic CRPC samples compared to primary tumours (Stylianou et al., 2019). Hypoxia-mediated stress in cancer cells following androgen-deprivation is also proposed to be an inducer of EMT in tumour cells (Byrne et al., 2016). Treatment of LNCaP xenograft tumours with bicalutamide led to a decrease in tumour oxygenation by 24 h, as well as a decrease in tumour vasculature on day 7, and changes in gene expression consistent with EMT. Consistent with this observation, growing LNCaP cells *in vitro* under hypoxic conditions increased expression of selected EMT-related genes (*IGF1, ITGA2* and *TIMP1*) after 24 h of treatment (Byrne et al., 2016). Hypoxia is a common feature in tumours and can lead to many changes in gene expression (McKeown, 2014; Fraga et al., 2015), and is a strong inducer of EMP (Cooke et al., 2012; Cursons et al., 2015; Azimi et al., 2017).

Molecular profiles of CTCs shed into the blood can help us acquire a better understanding of the relationships between castration, EMP and metastatic progression. The presence of mesenchymal CTCs has been previously associated with shorter time to castration-resistance as compared to those patients with predominantly epithelial CTCs (Yang et al., 2019). Our study comprised of molecular analysis of CTCs in four prostate cancer PDX models post-castration in comparison with non-castrated mice. CTCs were detected less often in castrated mice as compared to non-castrated mice in all models. Importantly, the numbers of CTCs per mL blood were similar between the castrated and non-castrated mice for all the models when CTCs were present. This was unexpected, as we had hypothesised that PDX-bearing mice that had responded to castration with tumour regression would shed fewer CTCs in the blood as compared to non-castrated mice. In clinical studies, it has been shown that baseline CTC counts correlate with poor therapy prognosis in patients responding to ADT ± orteronel, a cyp-17 inhibitor, however CTC numbers after treatment were not monitored (Goldkorn et al., 2021). In metastatic CRPC, it has been observed that baseline CTC numbers of more than five CTCs per 7.5 ml of blood correlate with poor overall survival and CTC enumeration post-treatment showed superior ability to serum PSA readings at predicting overall survival (De Bono et al., 2008).

Gene expression profiling of CTCs using our 42-gene panel and unsupervised hierarchical clustering in non-castrated mice (Hassan et al., 2021b) revealed three distinct molecular groups - 1) predominately mesenchymal gene expression, 2) predominantly epithelial gene expression, and 3) EMT hybrid phenotype with elevated expression of both epithelial and mesenchymal markers. The mesenchymal cluster had high expression of most mesenchymal markers and low or no expression of epithelial markers (including *KLK3*, which is specific for prostate). By contrast, the cluster with epithelial gene expression pattern had high expression for most epithelial associated genes and no or low expression of mesenchymal associated genes other than *FN1*. *FN1* encodes fibronectin, a protein known to protect cells from apoptosis (Fornaro et al., 2003), thus high expression of *FN1* in both epithelial and mesenchymal clusters may be related to this activity. In the mesenchymal cluster, *VIM*, a commonly studied mesenchymal-associated factor, had highest gene expression level out of all genes, and *EPCAM*, an epithelial-associated factor, had detectable expression in only one of these samples.

CTC enumeration has been shown to be associated with tumour size (Ruscetti et al., 2015; Lemech et al., 2016), progression free survival (Payne et al., 2012; Zhang and Armstrong, 2016; Wang et al., 2017; He et al., 2019) and overall survival (Lemech et al., 2016; Wang et al., 2017) in various clinical studies. Although we observed a relationship between estimated CTC enumeration and tumour size in our previous preclinical study of the same prostate cancer PDX models in non-castrated mice (Hassan et al., 2021b), no significant correlation was observed in this study of castrated mice. This is due to the high numbers of CTCs detected in the blood of castrated mice bearing regressed tumours. In some cases (24/37), CTCs were detected in the blood despite no measurable primary tumour following castration. This suggests that even though the tumour had regressed in response to castration, CTCs were being shed into the blood from the small tumour remnant at the initial implantation site and/or from undetected micrometastatic deposits.

CTCs were detected in the absence of measurable serum PSA in castrated mice. Indeed, an estimated 1–458 CTCs per mL of blood were present despite serum PSA levels being below detection for most castrated mice. Clinical studies have provided evidence of CTCs in prostate cancer patients responding to ADT, where CTC enumeration correlated with disease-free survival (Broncy and Paterlini-Bréchot, 2019). However, these results need independent validation in larger cohorts, since most studies in prostate cancer concentrate on patients with metastatic and/or CRPC. Furthermore, most studies in the context of localised prostate cancer focus on *KLK3* gene expression in CTCs. Our study shows downregulation of *KLK3* in all 4 PDX models and emphasises the importance of using a larger panel of genes to study CTCs as dependency on *KLK3* in this study would have led to many false negatives. So far there has been a study in localised prostate cancer patients that used CTC RNA scoring with an 8-gene panel and a droplet digital PCR assay. None of these patients had detectable CTC RNA scores using the PCR alone but whole genome amplification (WGA) prior to PCR resulted in CTC signal associated with pathological evidence of cancer cell dissemination (Miyamoto et al., 2018). Since CTC numbers are generally low in patients with localised disease as compared to those with metastatic cancers, highly sensitive technologies are necessary in this setting.

While we saw evidence of pronounced mesenchymal and hybrid gene expression clusters in the analysis of CTCs in castrated mice, we did not find evidence of the epithelial-like cluster (Figure 6). This is despite the immunofluorescence analysis of CTCs showing that epithelial CTCs (defined as KRT^+^VIM^−^) comprised over 50% of CTCs in 4 of 13 specimens (Tables 1, 2). The use of a panel of epithelial and mesenchymal transcripts compared to 2 protein markers likely accounts for this discrepancy and emphasises the challenges associated with the definition of epithelial or mesenchymal states for carcinoma cells. The epithelial cluster identified on the heat map only comprised of CTCs from non-castrated mice, and LuCaP96 was particularly prominent in this cluster. Expression of *KLK3* in this cluster was high compared to the mesenchymal cluster. These data reinforce a shift towards mesenchymal phenotype in CTCs after castration, consistent with the studies described above. While we have previously observed lower levels of CTC *KLK3* gene expression compared to primary tumours in all PDX models in non-castrated mice (Hassan et al., 2021b), *KLK3* expression was similar in CTC and tumour samples from castrated mice. This could be due to generally low *KLK3* expression in tumours in response to castration.

Several samples clustering closely together had very high expression of both epithelial and mesenchymal-associated genes. Such hybrid CTCs have been previously reported to be more aggressive in nature than epithelial or mesenchymal CTCs (Yu et al., 2013; Jolly et al., 2015; Comaills et al., 2016; Jolly et al., 2018; Saxena et al., 2019; Sun et al., 2019). While these published studies specifically looked at combined expression of epithelial and mesenchymal markers in individual CTCs, whether as single CTCs or in CTC clusters, in the current study it cannot be ruled out that this dual phenotypic expression could also indicate the presence of both epithelial and mesenchymal CTCs simultaneously in blood as our RT-qPCR analysis is a pooled analysis. However, since 44% of these samples had an estimated number of 1–3 CTCs per mL of blood, and having analysed only approximately 0.5 ml of blood, it is unlikely that the signature is from a mixed population in these cases. Samples with higher CTC numbers in our hybrid clusters nonetheless require immunocytochemical analyses to unequivocally establish the hybrid nature of these CTCs. Indeed, our immunocytochemical analysis of blood samples from castrated mice using vimentin and epithelial cytokeratins showed the presence of hybrid CTCs in all blood samples assessed (*n* = 13; Table 1).

The transcriptional regulation of *CDH1* expression by androgens is complex and dependent on an accessory factors and cellular context (Lin and Chuu, 2016). AR-mediated downregulation has been shown in prostate cancer cells when the AR is expressed in prostate cancer cells that do not usually express the receptor, but not in AR-expressing LNCaP cells (Nightingale et al., 2003). Loss of *CDH1* expression is a well-established hallmark of EMT (Fang and Kang, 2021). In our samples from castrated and non-castrated mice with mesenchymal gene expression (Figure 6), we observed relatively low levels of *CDH1*. Indeed, in most of these samples (7/11), *CDH1* expression was below the detection threshold.

The mesenchymal cluster included a stem-cell like profile with upregulation of *CD44* and downregulation of *CD24*, which is consistent with the simultaneous presence of EMT and stem-like features of tumour cells associated with metastatic potential of cancer cells (Mani et al., 2008; Ishiwata, 2016). Knockdown of *CDH1* in prostate cancer cell line PC-3 caused increased expression of the *CD44* gene (Deep et al., 2014), as initially reported in breast cancer cells (Mani et al., 2008). Furthermore, *CD44* expression has been associated with down-regulation of epithelial genes coupled with upregulation of mesenchymal genes in patients that have failed ADT (Shang et al., 2015). This pattern was absent in the epithelial cell cluster seen in non-castrated mice (Hassan et al., 2021b). CD44^High^CD24^Low^ is a stem cell feature (Palapattu et al., 2009), and presence of CD44^High^CD24^Low^ cells has been predictive of poorer prognosis in prostate cancer (Hurt et al., 2008). There is substantial evidence that EMP and presence of cancer stem cells plays a pivotal role in therapy resistance in prostate and other cancers (Ishiwata, 2016). Samples in the hybrid cluster also had low expression of *CD24* and high expression of *CD44* stem cell markers. Hybrid cells have been previously reported to be enriched in CSC-like phenotype and exhibit self-renewal capacity (Grosse-Wilde et al., 2015; Forte et al., 2017; Denisov and Perelmuter, 2018). Further investigation of these pathways may provide a better understanding of disease progression and uncover novel therapeutic targets.

After initiation of ADT in prostate cancer patients, EMT features were found to be increased in tumour cells (Sun et al., 2012; Laudato et al., 2019). In our study, CTC samples from both castrated and non-castrated mice were present in the mesenchymal cluster, suggesting that the mesenchymal state is conserved in the castrate CTCs. Moreover, CTCs from castrated mice were not observed in the epithelial cluster. These observations support the concept that castration can promote the induction of EMT, however EMT can also be induced in the absence of castration, indicating 1) the presence of non-castration-related mechanisms controlling EMP and 2) the complexity of this process. While the LuCaP96 model did not show significant gene expression changes in CTCs in the absence of androgen ablation, it showed a much higher degree of change after castration, further suggesting that castrate-driven CTCs have a specific pathway to EMP and CTC mobilisation.

We also observed an increased expression of *HIF1A*, a hypoxia related gene, in our mesenchymal cluster, as compared to the epithelial cluster. Hypoxia in the primary xenograft can be induced during androgen deprivation and may result in the induction of EMT (Byrne et al., 2016). The combination of ADT and hypoxia can induce adaptive androgen receptor signalling and/or development of AR-independent cancer cells (Geng et al., 2018). This could be attributed to hypoxia-induced expression of genes associated with stemness and EMT in prostate cancer cells (O’Reilly et al., 2019). Under hypoxic conditions, EMT-like changes including downregulation of E-cadherin, upregulation of Snail, and Wnt pathway activation were observed in various epithelial cancer cell lines (Cannito et al., 2008). Hypoxia-induced cellular plasticity and heterogeneity has been demonstrated in normal human mammary epithelial cells (Dhawan et al., 2016) and hypoxia is a well-established driver of EMT in breast cancer models (Cooke et al., 2012; Cursons et al., 2015).

Upregulation of *EGFR* has been associated with prostate cancer progression and metastasis using various prostate cancer cell line model systems (DU145, PC-3, LNCaP and C4-2B) (Gan et al., 2010; Day et al., 2017). Primary tumour EGFR overexpression correlated with migratory and metastatic phenotype of tumour cells in prostate cancer patients (Nastały et al., 2020). While CTCs were detected in only 13% (5/39) of high-risk patients in this study, all CTCs were positive for EGFR using immunocytochemistry (Nastały et al., 2020). By contrast we observed very low expression of *EGFR* in all three clusters using our RT-qPCR approach, although protein analysis is required to enable comparison to the previously published results.

Estrogens play a role in promoting prostate cancer progression (Bonkhoff and Berges, 2009) and estrogen receptor alpha (ERα) overexpression has been found in prostate during tumour formation (Bonkhoff and Berges, 2009). Furthermore, estrogen administration alongside testosterone increased incidence of prostate cancer in naïve mice to 100%, compared to 35–40% in mice that were only administered testosterone (Ricke et al., 2008). Our hybrid cluster comprises 5/8 samples that have very high *ESR1* expression, although the other three samples in this cluster lacked detectable *ESR1* expression.

PDX models provide a clinically relevant avenue to study cancer disease and progression. We used four different PDX models. LuCaP96 in particular demonstrated some unique features that are highly relevant to this study. LuCaP96 was established from a Gleason score 5+4 primary tumour of a patient undergoing androgen ablation therapy using tissue collected 1 month prior to clinical documentation of castration-resistant disease (Nguyen et al., 2017). By contrast, the other PDXs in this study were established using tissue from metastatic deposits (McCulloch et al., 2005; Nguyen et al., 2017). LuCaP70 and LuCaP105 were established from patients with CRPC (liver and bone metastasis, respectively), while BM18 was established from a bone metastasis obtained from a treatment naïve donor. In our study, LuCaP96 had the most epithelial-like gene expression profile, potentially reflecting its derivation from a primary tumour. Upon unsupervised hierarchical clustering of all castrated and non-castrated blood samples, the majority of samples in the epithelial cluster were from LuCaP96 non-castrated mice (5/7; 71%). By contrast, in each of the hybrid and mesenchymal clusters there was only 1 LuCaP96 sample–both of which were from castrated mice. When LuCaP96 castrated and non-castrated samples were compared, 19/41 genes in CTCs and 5/41 genes in primary tumours were significantly different, with genes *APLN* and *CD44* being significantly different in both comparisons. Thus the effects of castration on this model were pronounced, despite the variable effect of castration on tumour growth. On comparing CTC gene expression of all non-castrated mice with samples from castrated mice that had regressed tumours, irrespective of PDX type, no significant difference in gene expression was observed. Similarly, only three genes were significantly different between CTC gene expression in non-castrated and castrated (non-regressed) mice (*GRHL2, CD44* and *ILK*). The low variation observed between both groups in this pooled analysis could be attributed to high variation between samples and PDX models, which is being masked when all the samples are analysed together.

CTC enumeration predicts poor prognosis earlier than serum PSA levels in prostate cancer patients (Onstenk et al., 2016), and our study also found the presence of residual disease in the blood using a CTC assay despite no detectable PSA. Therefore, CTCs may be a particularly sensitive marker for residual disease. Molecular characterisation of CTCs has clinical potential to aid treatment decision-making (Broncy and Paterlini-Bréchot, 2019). One such recent study showed that expression of *AR-V7* of the *AR* in CTCs was correlated with poor patient response to abiraterone and enzalutamide therapy in metastatic prostate cancer (Sciarra et al., 2019), and thus CTC analyses may be a useful tool for the prediction of treatment response and to inform therapy decision making.

## Conclusion

Our study uncovered the unexpected presence of CTCs in mice in which the primary tumour was regressed, sometimes completely, providing a novel insight into the possible clinical potential of liquid biopsy in detecting occult disease after castration. This is especially important given that the commonly used biomarker PSA was undetectable in the blood of these mice, and CTC PSA expression was also very low/undetectable. We identified a shift from epithelial towards mesenchymal and hybrid phenotypes in CTCs from castrated mice compared to non-castrated mice. Our study suggests that CTCs are potentially a highly sensitive tool for early prediction of metastasis that appears more sensitive than serum PSA, although we had the advantage of using human-specific (and thus tumour-specific) RT-qPCR primers in the context of a murine background in the current study. Translating this observation to the clinical setting is complicated by the lack of general tumour cell/CTC-specific mRNAs that would allow accurate, sensitive detection of CTCs among nucleated cells in the blood. Molecular characterisation of CTCs provides a window towards developing a deeper understanding of the association between castration and tumour cell EMP, particularly the hybrid EMP state. This may in turn provide novel therapeutic opportunities that, when used alongside ADT, could help prevent the development of CRPC and metastasis by restricting tumour cell plasticity and motility, and thus dissemination.

## Data Availability

The raw data supporting the conclusions of this article will be made available by the authors, without undue reservation.
