# Supplementary material for: Circulating Tumour Cells Indicate the Presence of Residual Disease Post-Castration in Prostate Cancer Patient-Derived Xenograft Models
Source: Front Cell Dev Biol. 2022 Apr 13;10:858013. doi: 10.3389/fcell.2022.858013 (PMC9043137; doi:10.3389/fcell.2022.858013)
Supplement: Supplementary file 1 [file Presentation1.PPTX]

## Slide 1
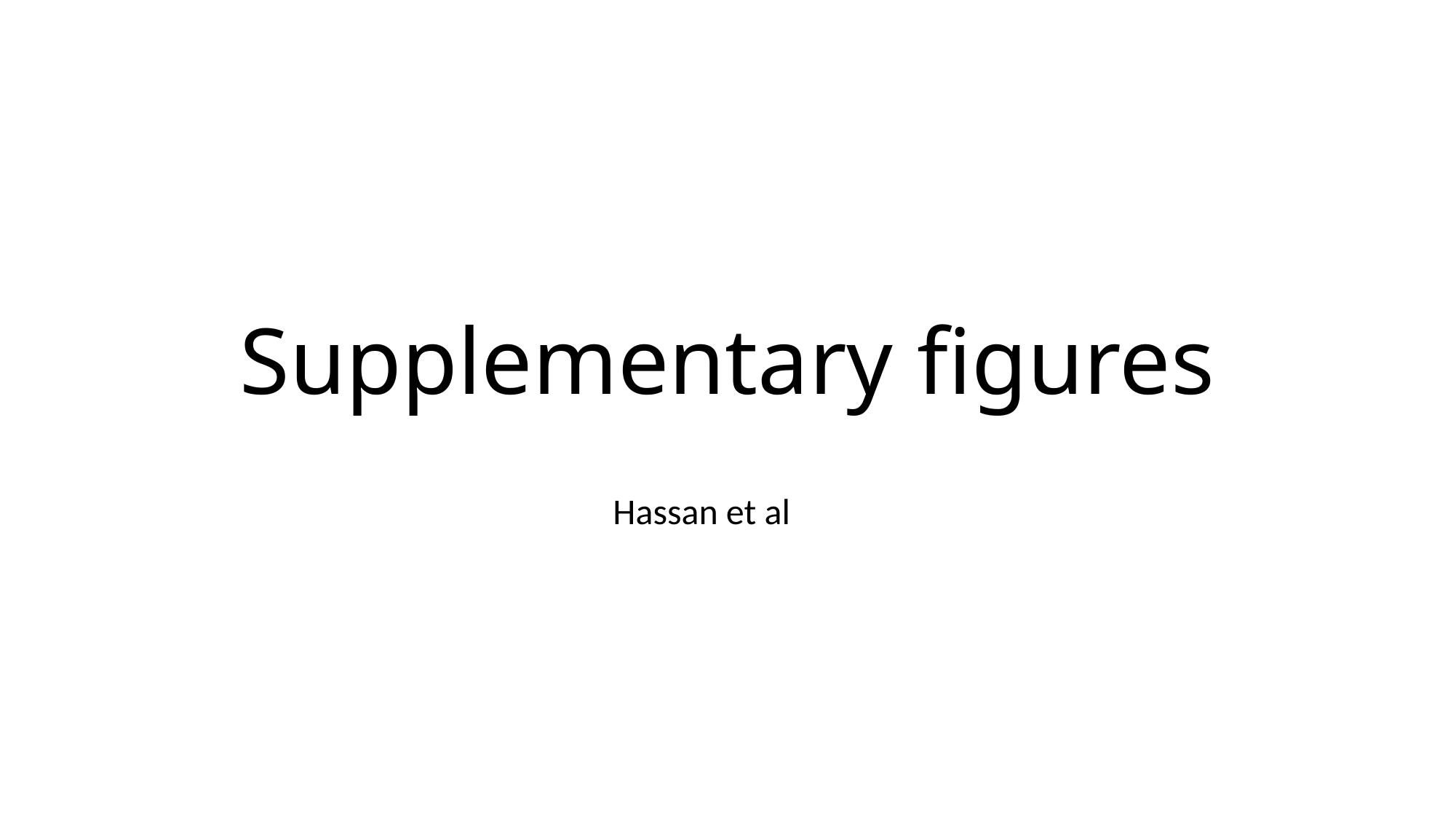

# Supplementary figures
Hassan et al

## Slide 2
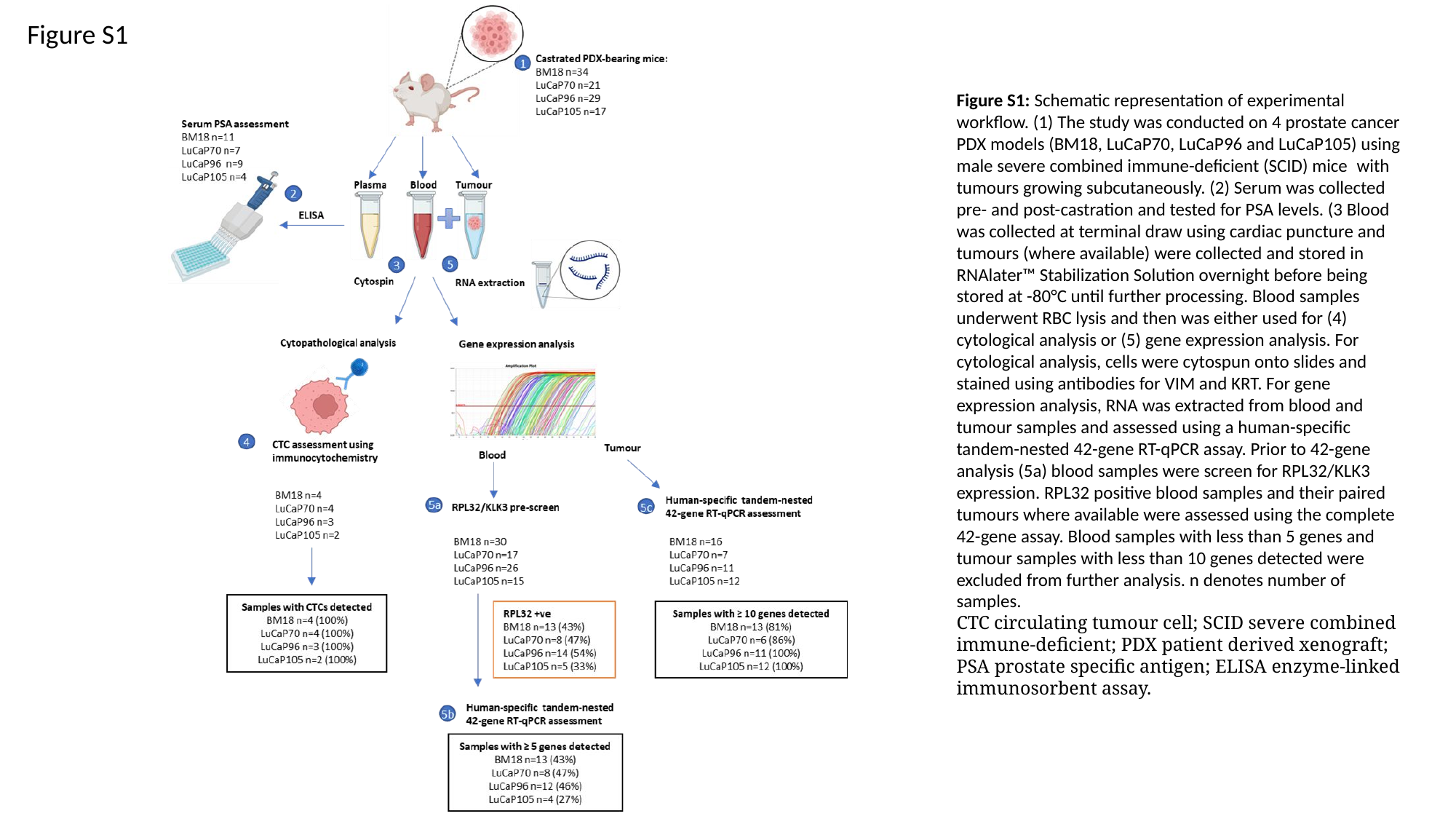

Figure S1
Figure S1: Schematic representation of experimental workflow. (1) The study was conducted on 4 prostate cancer PDX models (BM18, LuCaP70, LuCaP96 and LuCaP105) using male severe combined immune-deficient (SCID) mice with tumours growing subcutaneously. (2) Serum was collected pre- and post-castration and tested for PSA levels. (3 Blood was collected at terminal draw using cardiac puncture and tumours (where available) were collected and stored in RNAlater™ Stabilization Solution overnight before being stored at -80°C until further processing. Blood samples underwent RBC lysis and then was either used for (4) cytological analysis or (5) gene expression analysis. For cytological analysis, cells were cytospun onto slides and stained using antibodies for VIM and KRT. For gene expression analysis, RNA was extracted from blood and tumour samples and assessed using a human-specific tandem-nested 42-gene RT-qPCR assay. Prior to 42-gene analysis (5a) blood samples were screen for RPL32/KLK3 expression. RPL32 positive blood samples and their paired tumours where available were assessed using the complete 42-gene assay. Blood samples with less than 5 genes and tumour samples with less than 10 genes detected were excluded from further analysis. n denotes number of samples.
CTC circulating tumour cell; SCID severe combined immune-deficient; PDX patient derived xenograft; PSA prostate specific antigen; ELISA enzyme-linked immunosorbent assay.

## Slide 3
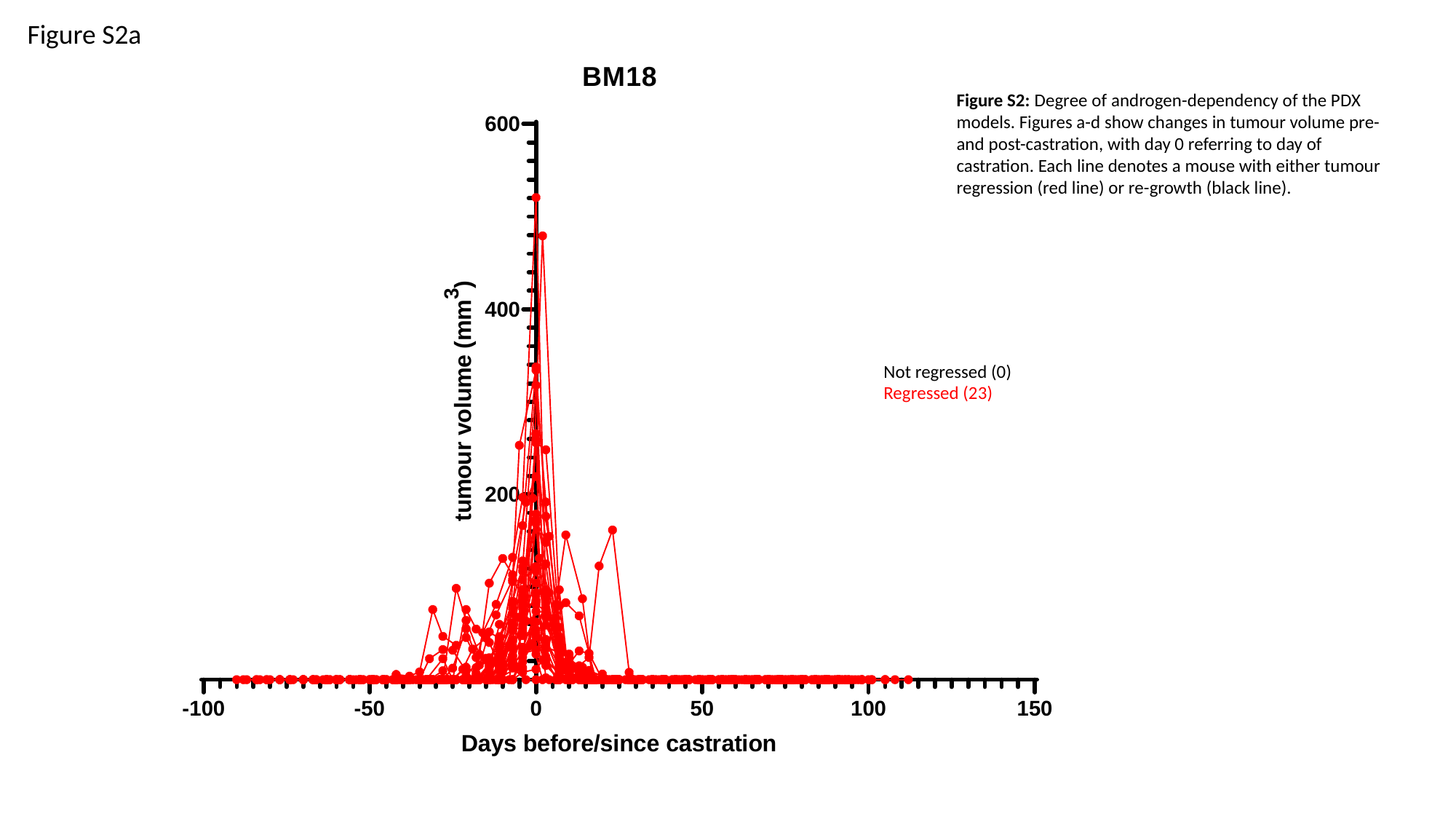

Figure S2a
Figure S2: Degree of androgen-dependency of the PDX models. Figures a-d show changes in tumour volume pre- and post-castration, with day 0 referring to day of castration. Each line denotes a mouse with either tumour regression (red line) or re-growth (black line).
Not regressed (0)
Regressed (23)

## Slide 4
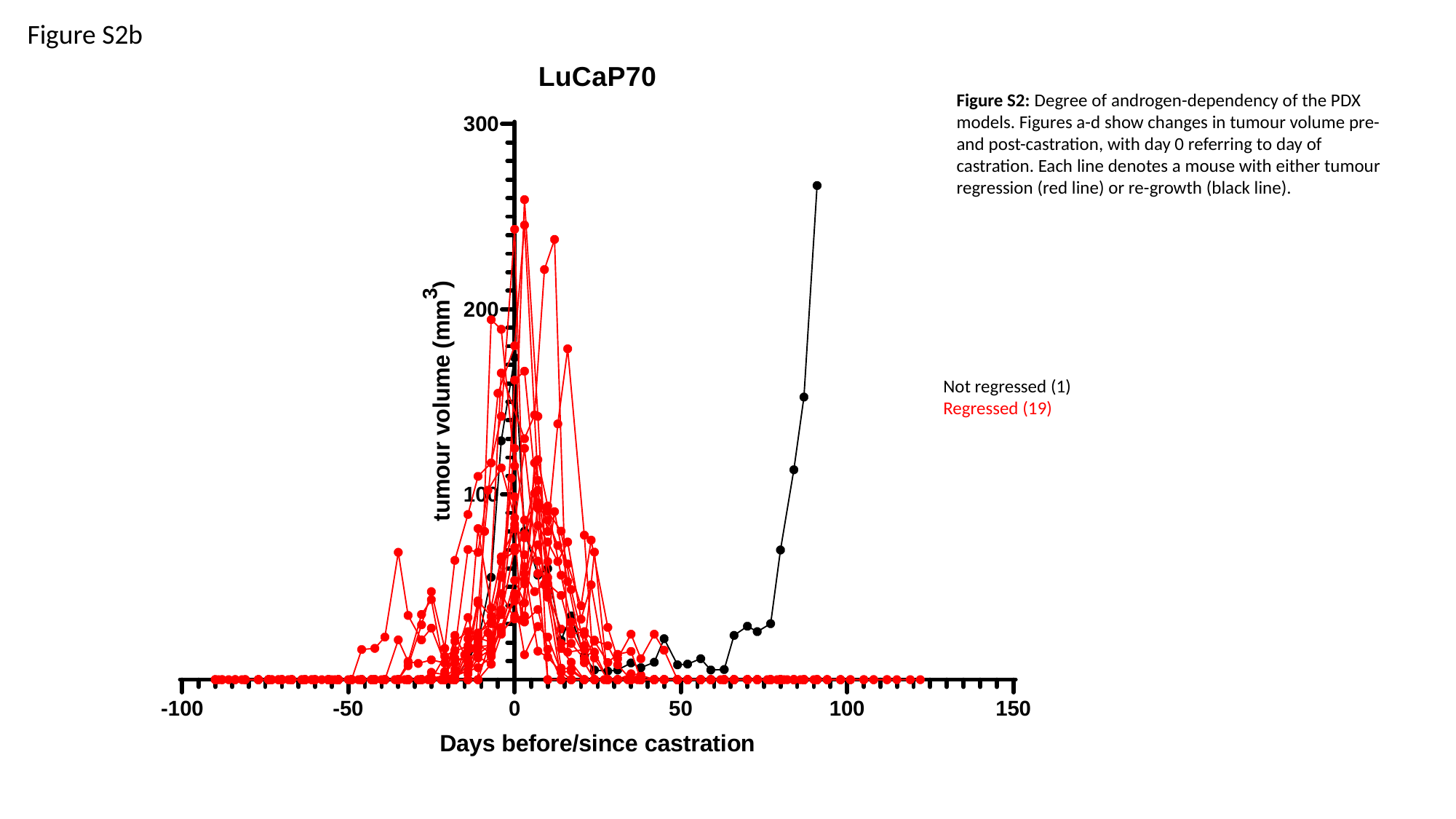

Figure S2b
Figure S2: Degree of androgen-dependency of the PDX models. Figures a-d show changes in tumour volume pre- and post-castration, with day 0 referring to day of castration. Each line denotes a mouse with either tumour regression (red line) or re-growth (black line).
Not regressed (1)
Regressed (19)

## Slide 5
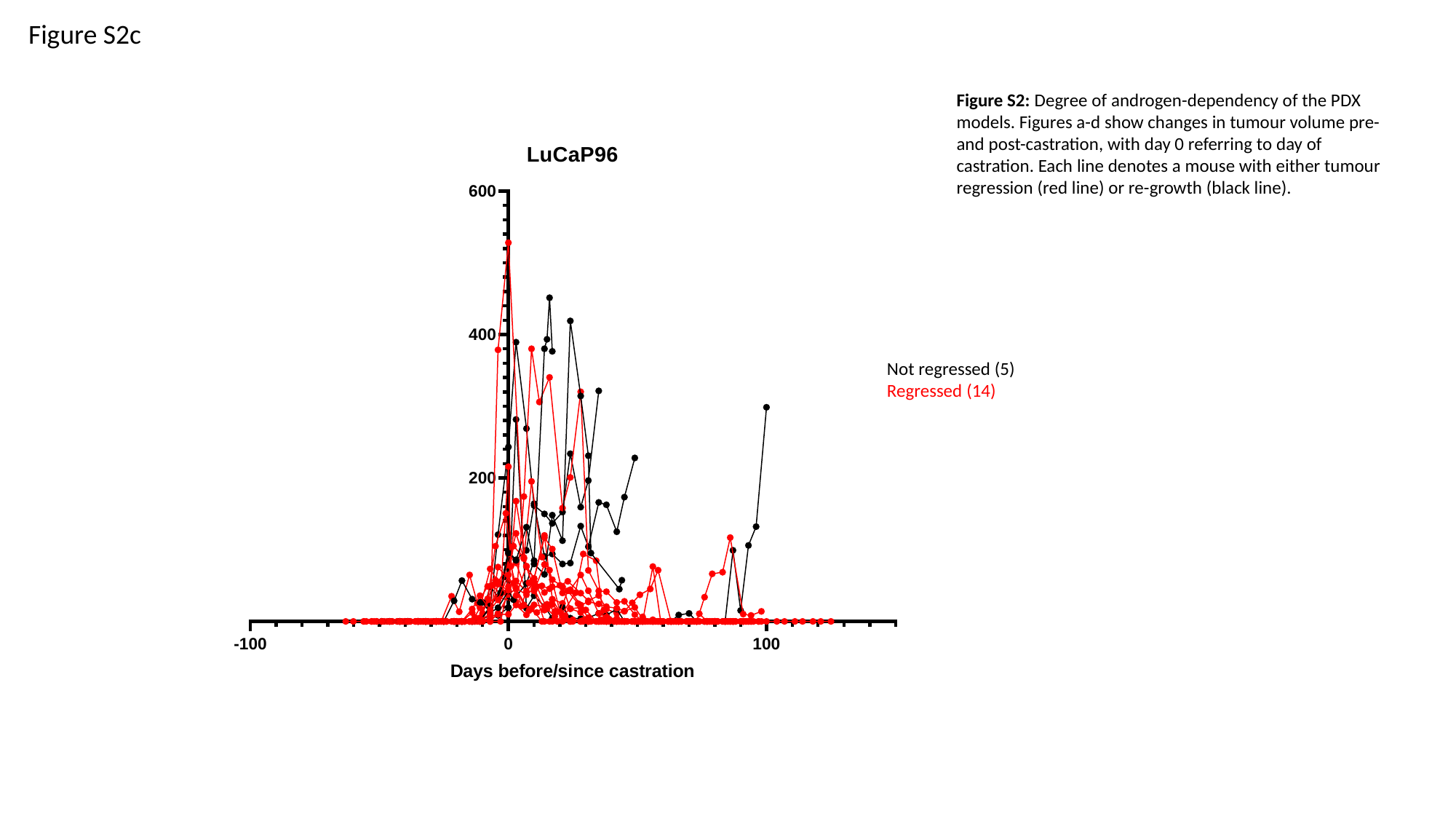

Figure S2c
Figure S2: Degree of androgen-dependency of the PDX models. Figures a-d show changes in tumour volume pre- and post-castration, with day 0 referring to day of castration. Each line denotes a mouse with either tumour regression (red line) or re-growth (black line).
Not regressed (5)
Regressed (14)

## Slide 6
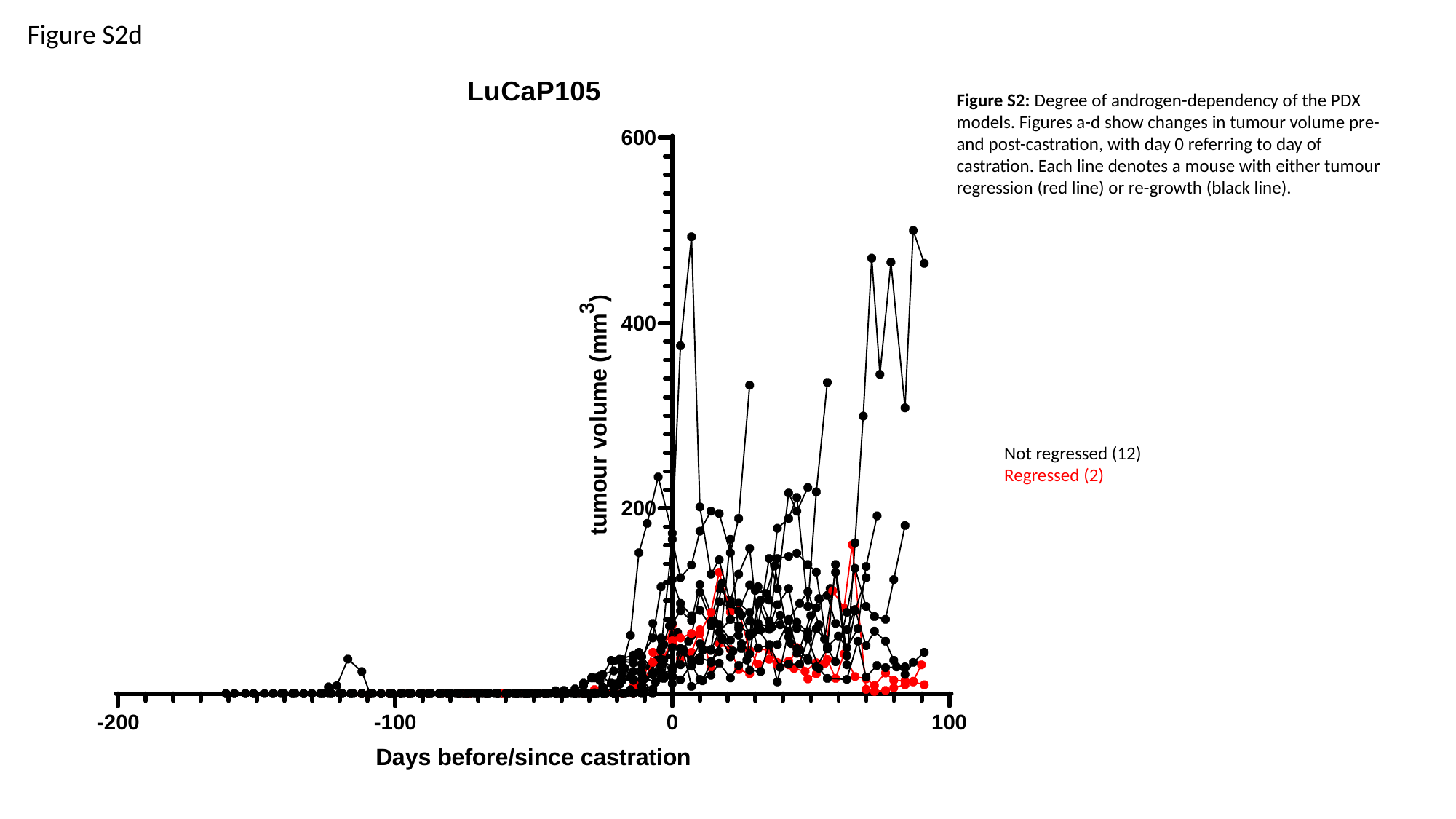

Figure S2d
Figure S2: Degree of androgen-dependency of the PDX models. Figures a-d show changes in tumour volume pre- and post-castration, with day 0 referring to day of castration. Each line denotes a mouse with either tumour regression (red line) or re-growth (black line).
Not regressed (12)
Regressed (2)

## Slide 7
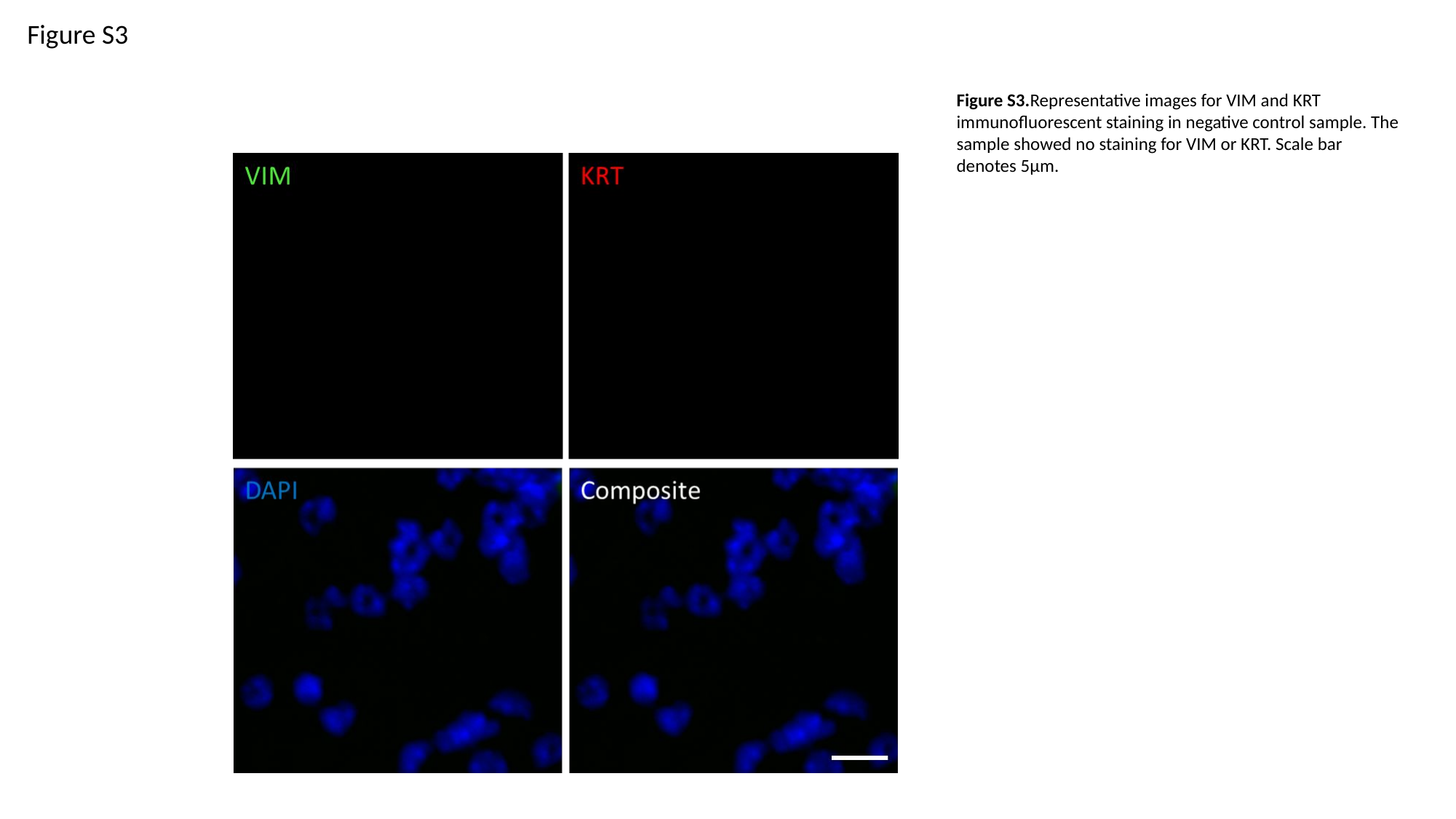

Figure S3
Figure S3.Representative images for VIM and KRT immunofluorescent staining in negative control sample. The sample showed no staining for VIM or KRT. Scale bar denotes 5µm.

## Slide 8
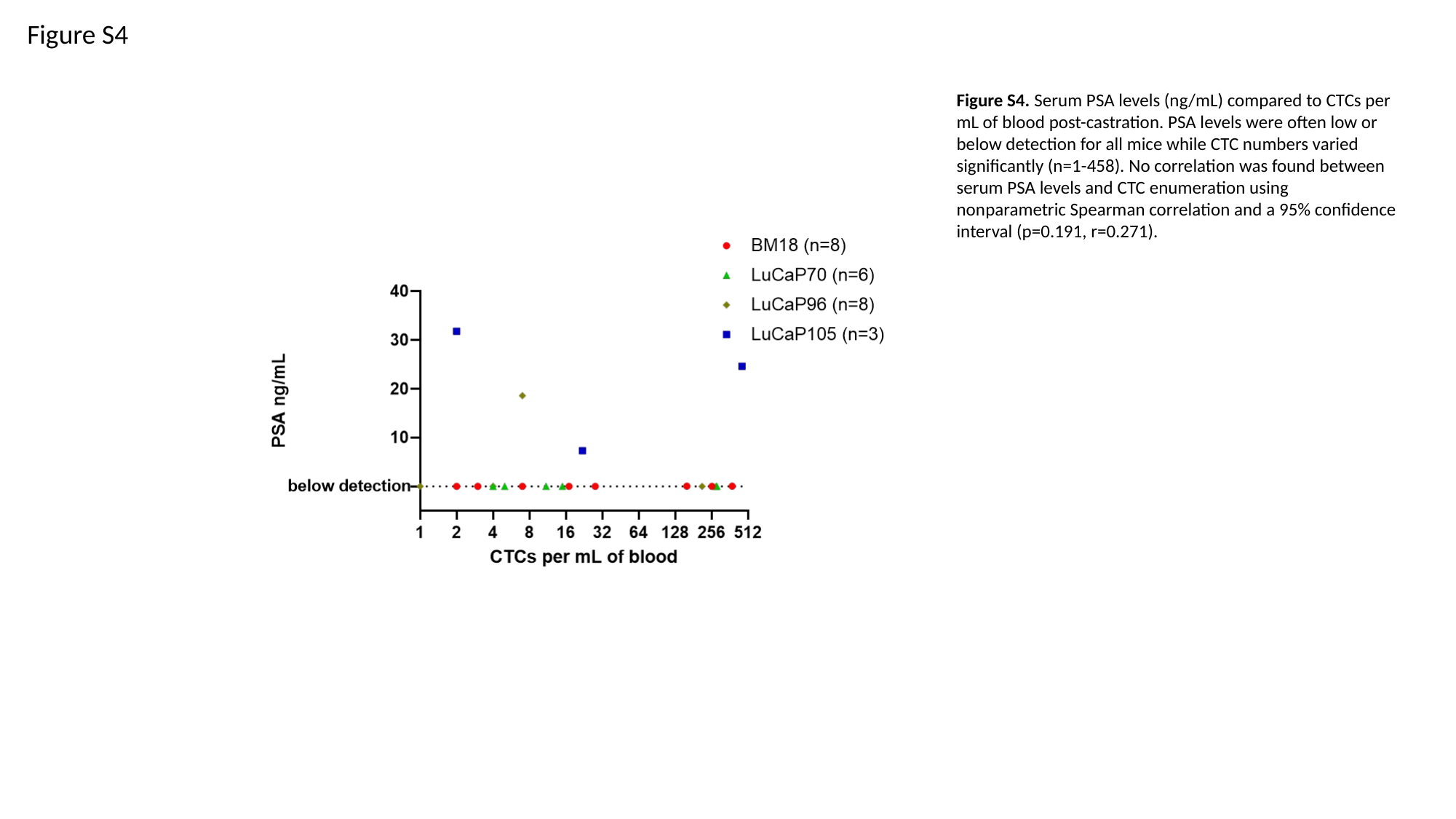

Figure S4
Figure S4. Serum PSA levels (ng/mL) compared to CTCs per mL of blood post-castration. PSA levels were often low or below detection for all mice while CTC numbers varied significantly (n=1-458). No correlation was found between serum PSA levels and CTC enumeration using nonparametric Spearman correlation and a 95% confidence interval (p=0.191, r=0.271).

## Slide 9
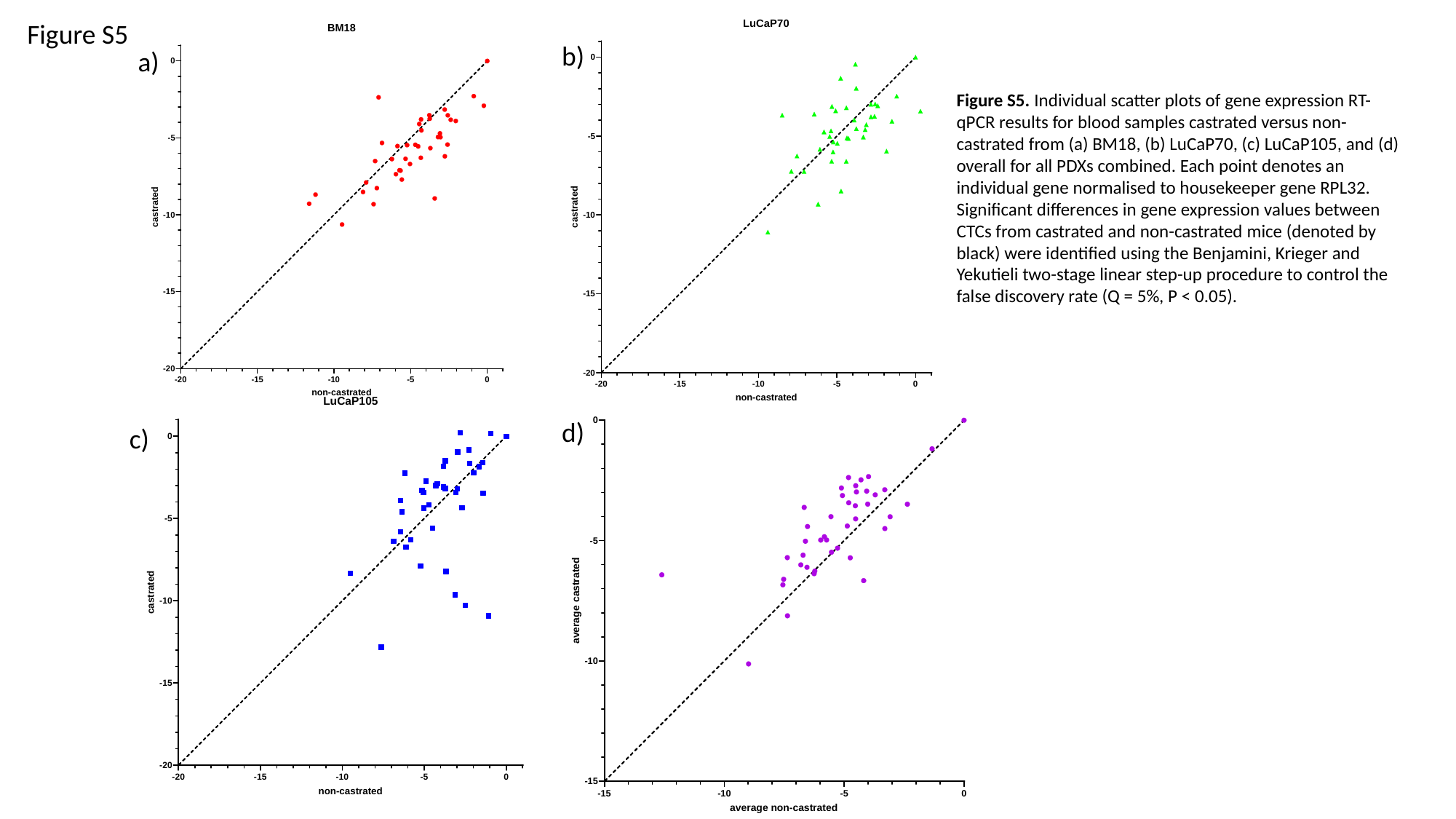

Figure S5
b)
a)
Figure S5. Individual scatter plots of gene expression RT-qPCR results for blood samples castrated versus non-castrated from (a) BM18, (b) LuCaP70, (c) LuCaP105, and (d) overall for all PDXs combined. Each point denotes an individual gene normalised to housekeeper gene RPL32. Significant differences in gene expression values between CTCs from castrated and non-castrated mice (denoted by black) were identified using the Benjamini, Krieger and Yekutieli two-stage linear step-up procedure to control the false discovery rate (Q = 5%, P < 0.05).
d)
c)

## Slide 10
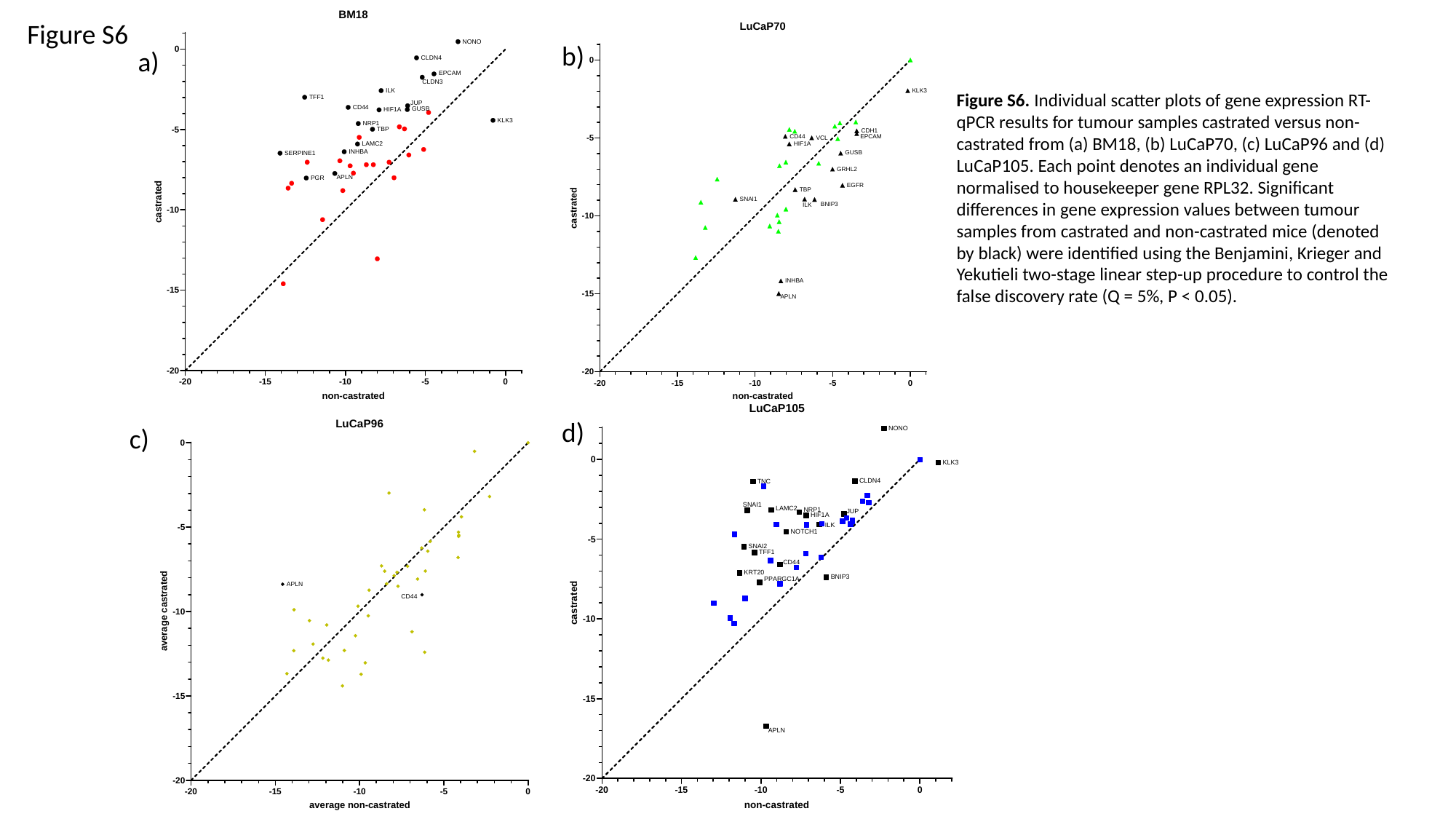

Figure S6
b)
a)
Figure S6. Individual scatter plots of gene expression RT-qPCR results for tumour samples castrated versus non-castrated from (a) BM18, (b) LuCaP70, (c) LuCaP96 and (d) LuCaP105. Each point denotes an individual gene normalised to housekeeper gene RPL32. Significant differences in gene expression values between tumour samples from castrated and non-castrated mice (denoted by black) were identified using the Benjamini, Krieger and Yekutieli two-stage linear step-up procedure to control the false discovery rate (Q = 5%, P < 0.05).
d)
c)

## Slide 11
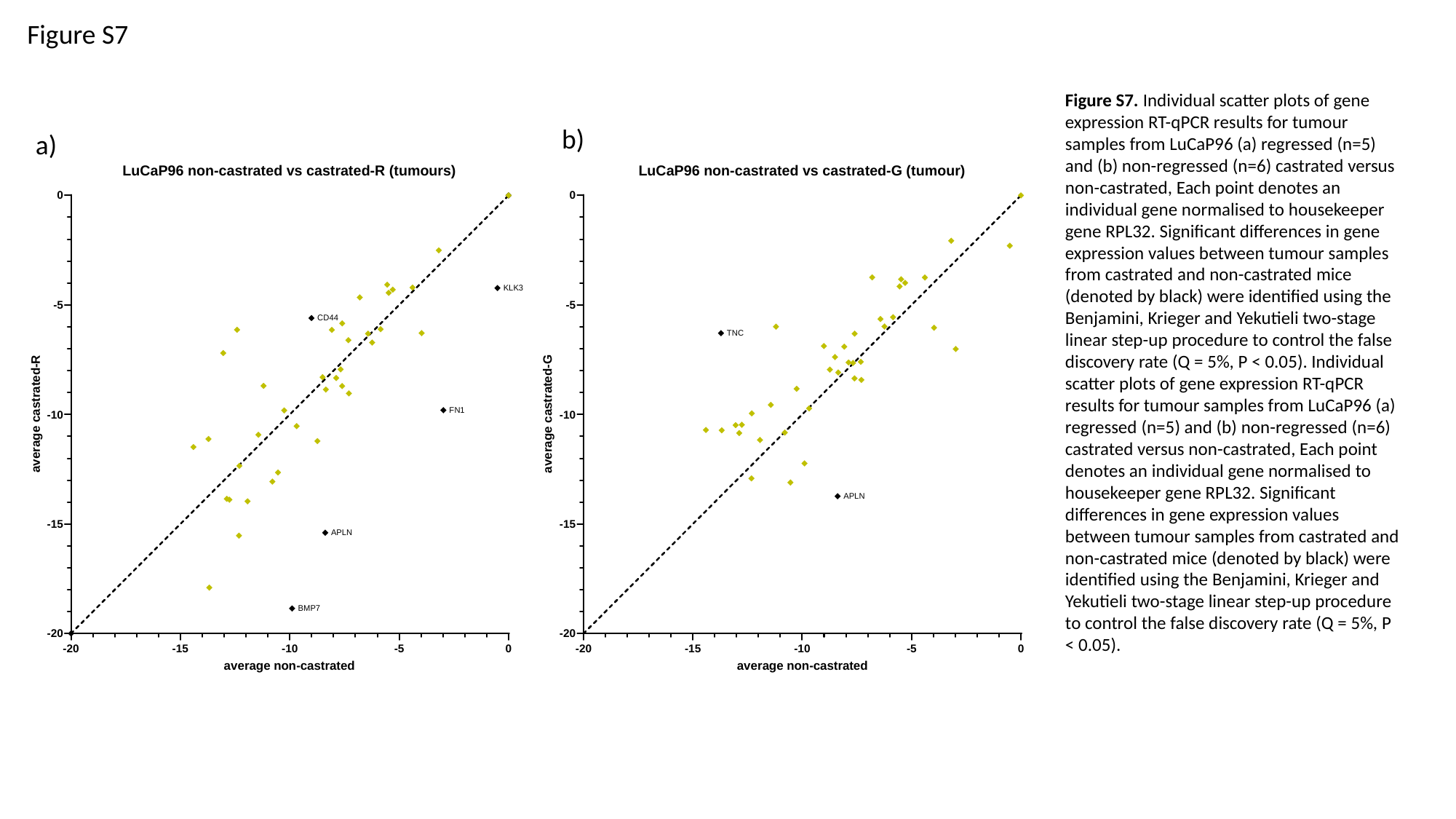

Figure S7
Figure S7. Individual scatter plots of gene expression RT-qPCR results for tumour samples from LuCaP96 (a) regressed (n=5) and (b) non-regressed (n=6) castrated versus non-castrated, Each point denotes an individual gene normalised to housekeeper gene RPL32. Significant differences in gene expression values between tumour samples from castrated and non-castrated mice (denoted by black) were identified using the Benjamini, Krieger and Yekutieli two-stage linear step-up procedure to control the false discovery rate (Q = 5%, P < 0.05). Individual scatter plots of gene expression RT-qPCR results for tumour samples from LuCaP96 (a) regressed (n=5) and (b) non-regressed (n=6) castrated versus non-castrated, Each point denotes an individual gene normalised to housekeeper gene RPL32. Significant differences in gene expression values between tumour samples from castrated and non-castrated mice (denoted by black) were identified using the Benjamini, Krieger and Yekutieli two-stage linear step-up procedure to control the false discovery rate (Q = 5%, P < 0.05).
b)
a)

## Slide 12
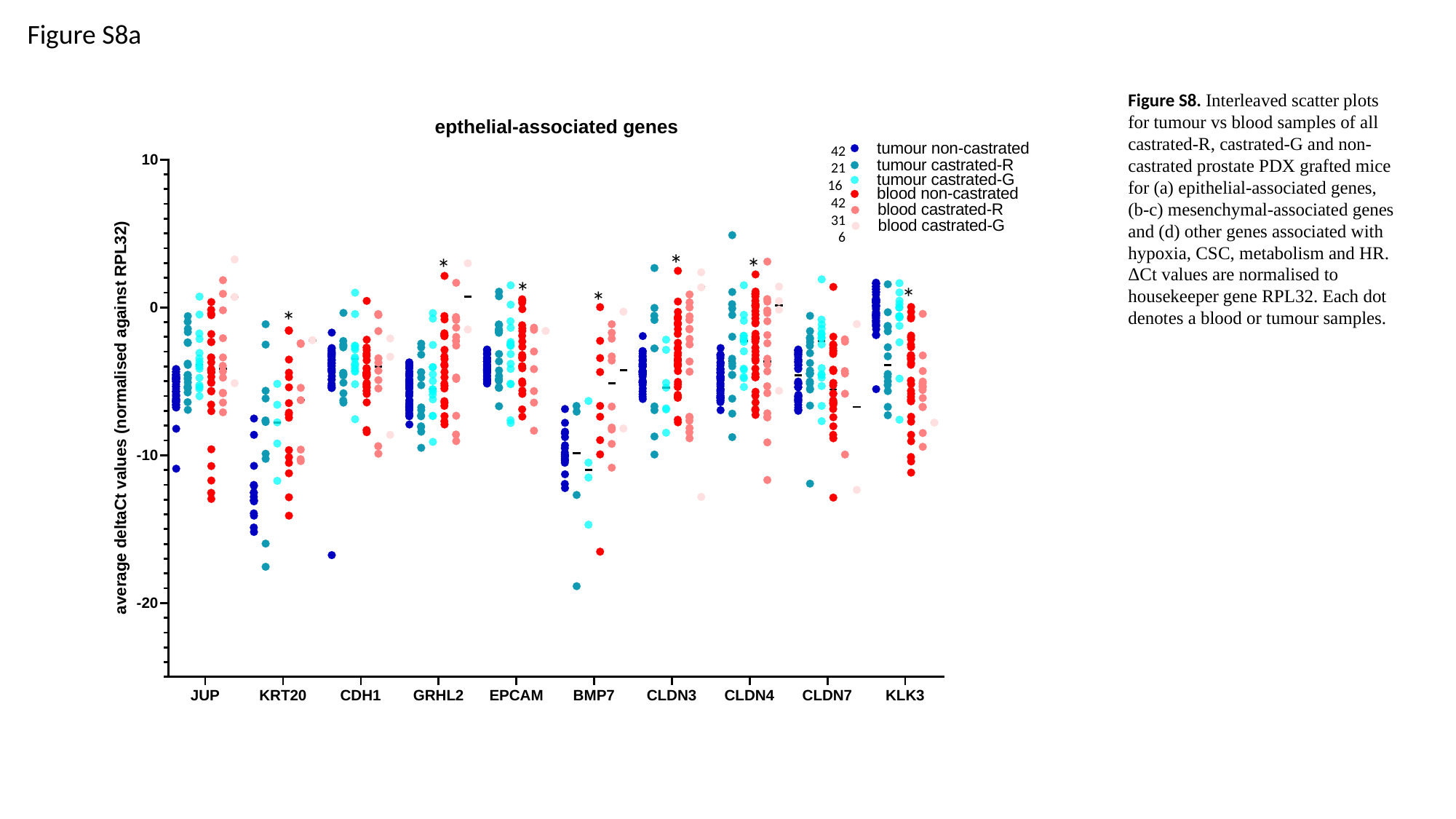

Figure S8a
Figure S8. Interleaved scatter plots for tumour vs blood samples of all castrated-R, castrated-G and non-castrated prostate PDX grafted mice for (a) epithelial-associated genes, (b-c) mesenchymal-associated genes and (d) other genes associated with hypoxia, CSC, metabolism and HR. ΔCt values are normalised to housekeeper gene RPL32. Each dot denotes a blood or tumour samples.
42
21
16
42
31
6
*
*
*
*
*
*
*

## Slide 13
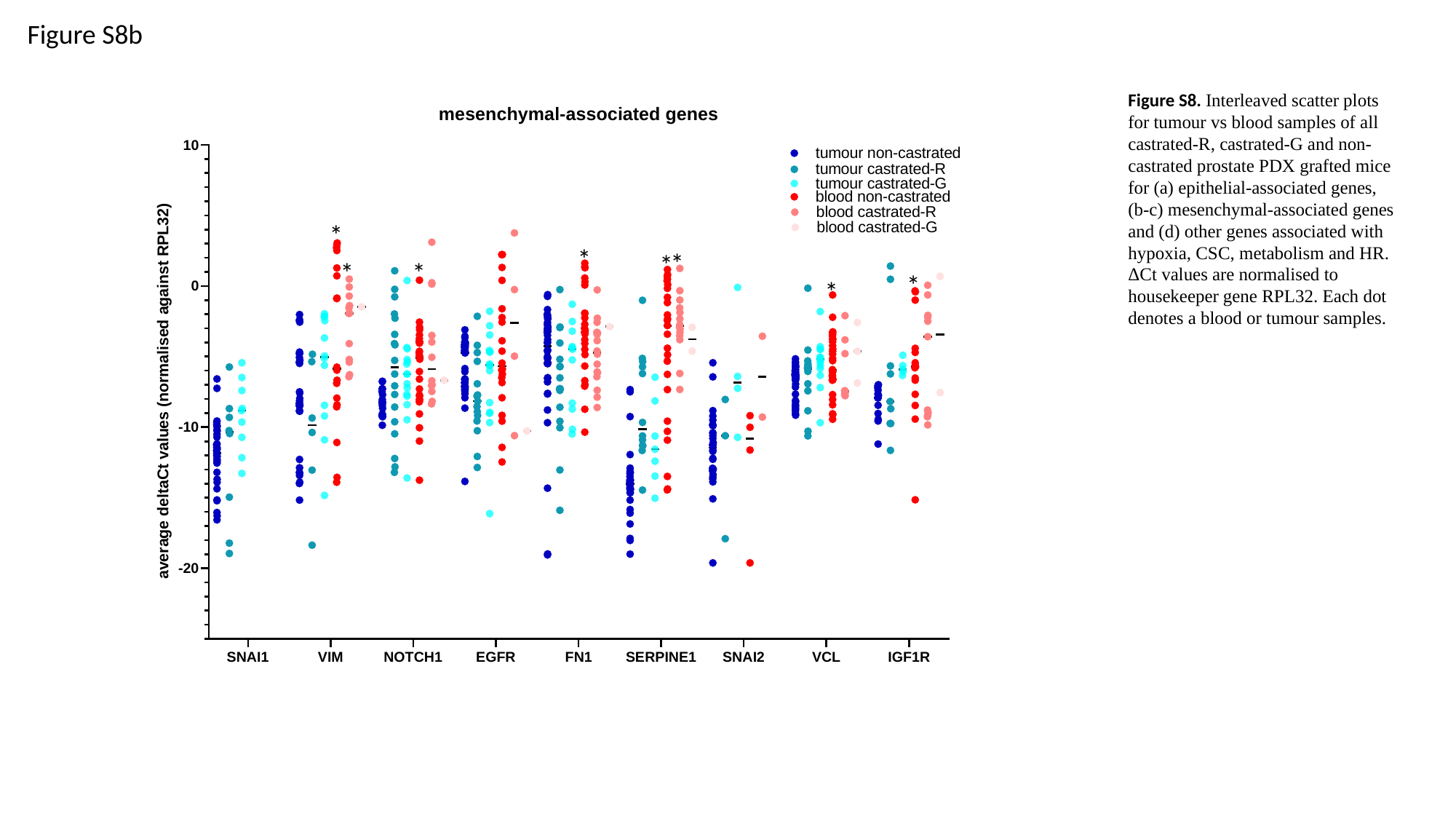

Figure S8b
Figure S8. Interleaved scatter plots for tumour vs blood samples of all castrated-R, castrated-G and non-castrated prostate PDX grafted mice for (a) epithelial-associated genes, (b-c) mesenchymal-associated genes and (d) other genes associated with hypoxia, CSC, metabolism and HR. ΔCt values are normalised to housekeeper gene RPL32. Each dot denotes a blood or tumour samples.
*
*
*
*
*
*
*
*

## Slide 14
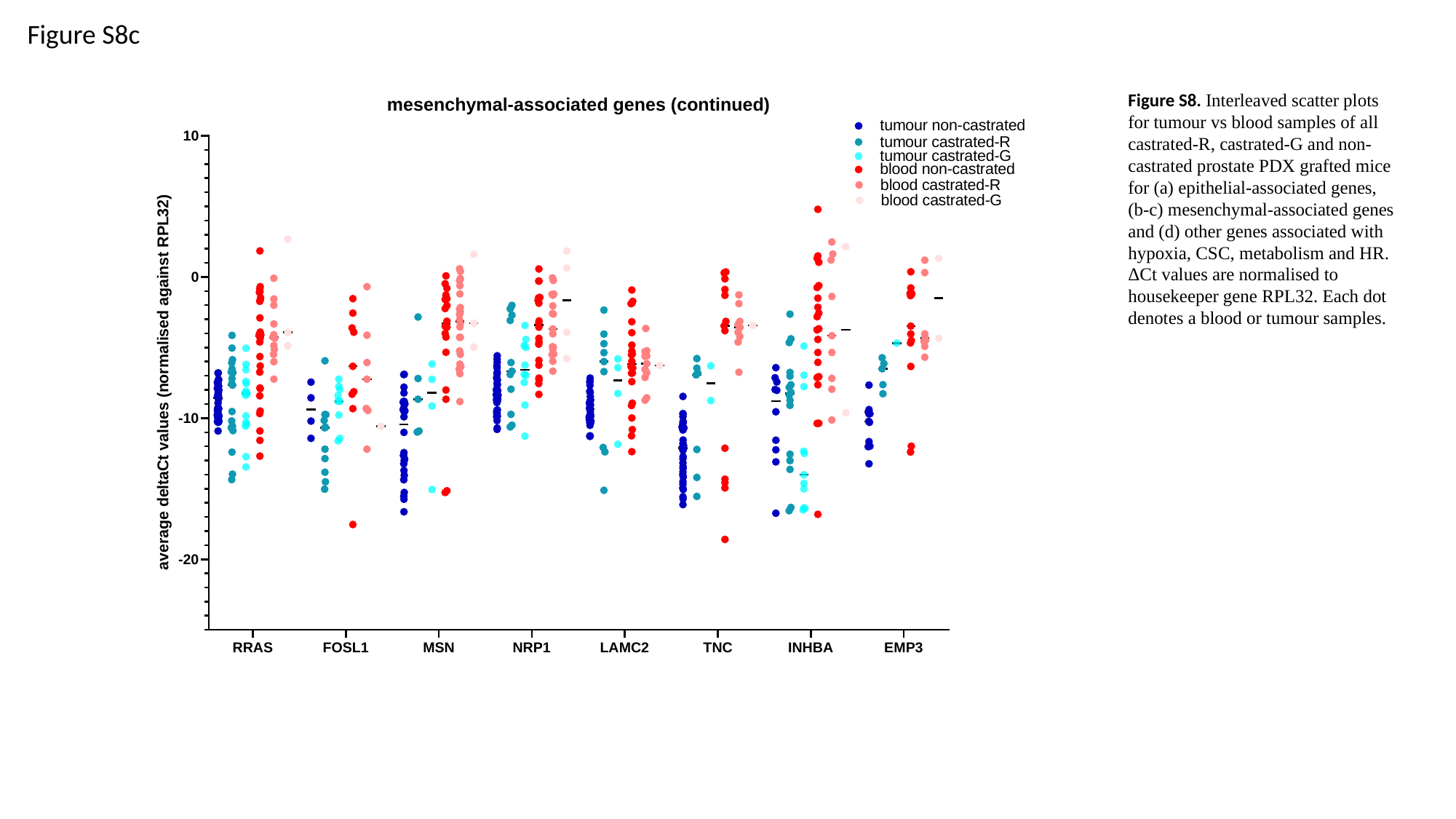

Figure S8c
Figure S8. Interleaved scatter plots for tumour vs blood samples of all castrated-R, castrated-G and non-castrated prostate PDX grafted mice for (a) epithelial-associated genes, (b-c) mesenchymal-associated genes and (d) other genes associated with hypoxia, CSC, metabolism and HR. ΔCt values are normalised to housekeeper gene RPL32. Each dot denotes a blood or tumour samples.

## Slide 15
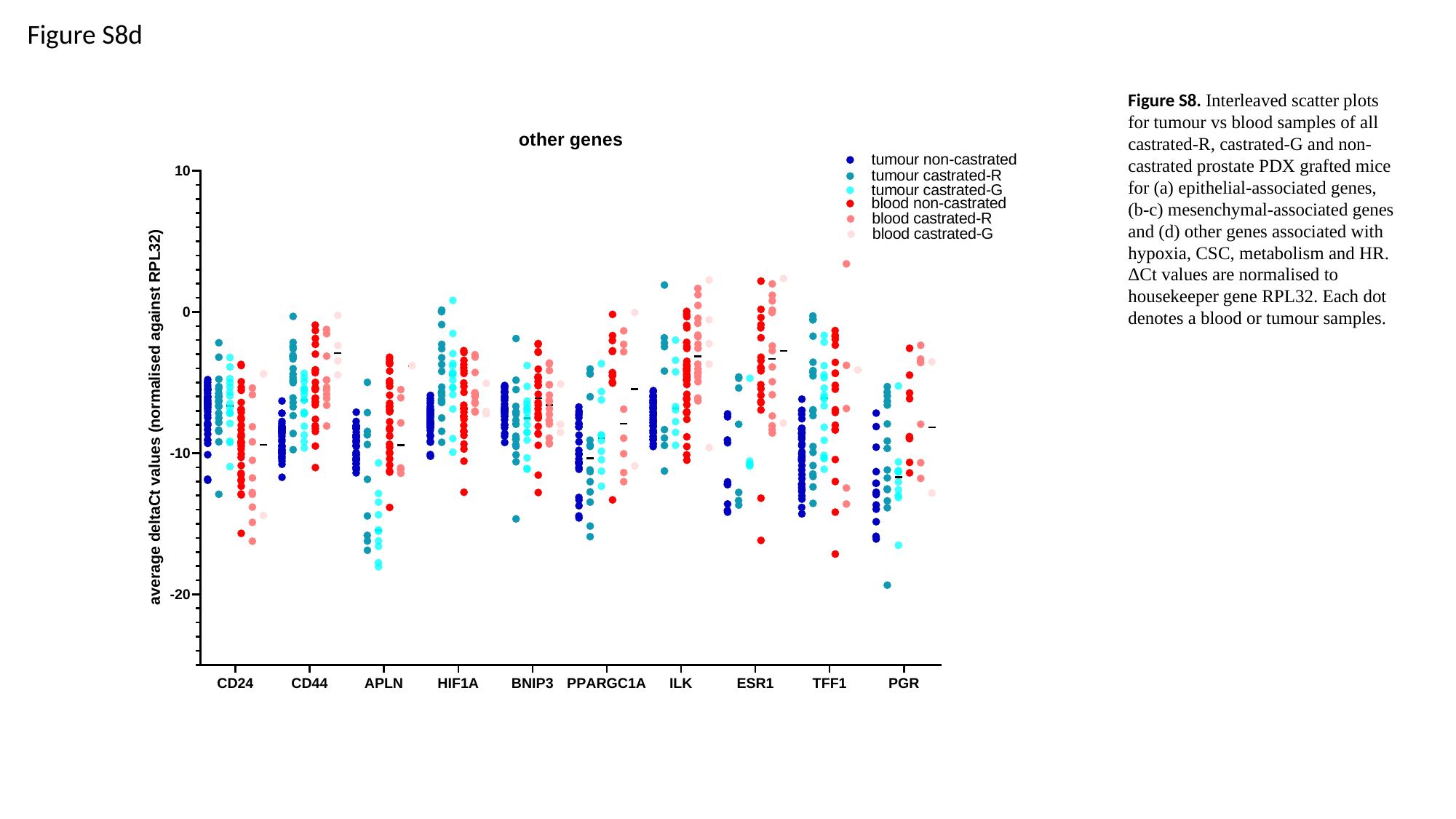

Figure S8d
Figure S8. Interleaved scatter plots for tumour vs blood samples of all castrated-R, castrated-G and non-castrated prostate PDX grafted mice for (a) epithelial-associated genes, (b-c) mesenchymal-associated genes and (d) other genes associated with hypoxia, CSC, metabolism and HR. ΔCt values are normalised to housekeeper gene RPL32. Each dot denotes a blood or tumour samples.
